# Advancing in vitro assessment of iodide uptake inhibition: integrating a novel biotransformation pretreatment step

**DOI:** 10.1007/s00204-025-04034-y

**Published:** 2025-05-12

**Authors:** Puja Kumari, Sebastian Lungu-Mitea, Jiří Novák, Klára Hilscherová

**Affiliations:** 1 Faculty of Science, RECETOX, Masaryk University, Kamenice 753/5, Pavilion A29, Kotlarska 2, 625 00 Brno, Czech Republic; 2 Department of Biology and Environmental Science, Linnaeus University, SE-39182 Kalmar, Sweden

**Keywords:** NIS, Thyroid hormone, Biotransformation, Sandell–Kolthoff reaction, SeqAPASS

## Abstract

Thyroid hormones (TH) are essential for vertebrate development, growth, and metabolism. The increasing prevalence of anthropogenic chemicals with TH-disrupting potential highlights the urgent need for advanced methods to assess their impact on TH homeostasis. Inhibition of the sodium–iodide symporter (NIS) has been identified as a key molecular initiating event disrupting the TH system across species, with significant relevance for diagnostic and therapeutic applications in various carcinomas. This study presents in vitro bioassays for evaluating the effects of compounds on iodide uptake into cells, a critical step in TH production mediated by NIS. Two novel stably transfected human cell lines overexpressing human NIS were employed along with a rat thyroid cell model FRTL-5, using colorimetric Sandell–Kolthoff (SK) reaction for iodide detection. The results from 23 model compounds demonstrate comparability across various in vitro models and radioactivity-based assays. To enhance physiological relevance, an external biotransformation system (BTS) was integrated and optimized for live-cell compatibility without inducing cytotoxicity or interfering with the assay. Compounds identified as NIS inhibitors were evaluated using the BTS-augmented assay, which revealed that metabolic activity mitigated the inhibitory effects of some chemicals. The augmented assay exhibited strong concordance with in vivo and in silico biotransformation data. Protein sequence alignment confirmed high conservation of NIS functional domains across vertebrates, reinforcing the cross-species applicability of the findings. The SK-based NIS assay, with optional BTS integration, represents a sensitive, robust, and high-throughput amendable alternative to radioactivity-based methods, for characterizing the impacts of individual compounds and complex environmental mixtures on TH homeostasis.

**Supplementary Information:**

The online version contains supplementary material available at 10.1007/s00204-025-04034-y.

## Introduction

The pervasive use of chemicals in pesticide mixtures, personal and household products, cosmetics, and other applications leads to constant, chronic exposure to humans and other biota. Some of these chemicals exhibit endocrine-disrupting potential (Jiang and Xiong 2016; McKinlay et al. 2008). Endocrine disruption involves chemical interference with hormone function, impacting crucial physiological processes such as growth, development, reproduction or metabolism (Ghassabian and Trasande 2018). Notably, some chemicals have been found to specifically disrupt thyroid hormone (TH) homeostasis (Tu et al. 2016; Xu et al. 2019). Thyroid hormone system disruption (THSD) significantly affects various species, including mammals, fish, and amphibians. For example, next to the multiple effects in mammals and humans discussed below, THSD can also lead to altered growth rates and impaired osmoregulation in fish (Nugegoda and Kibria 2017) and disrupt metamorphosis in amphibians (Miyata and Ose 2012).

The thyroid gland, responsible for releasing TH, plays a critical role in maintaining TH levels (Forner-Piquer et al. 2024; Tu et al. 2016). In humans, TH production begins around the 11th week of gestation and increases as the fetus develops. Maternal TH transfer through the placenta is vital for (neuro)development, and even slight alterations in TH levels can have long-term consequences (Woods et al. 2023). Although fetal development is the most sensitive period, TH play a crucial role throughout life, and TH dysregulation has been linked to numerous irreversible health issues, such as bipolar disorder, Alzheimer’s disease, and depression (Baksi and Pradhan 2021).

As the interference with TH production, transport, and signaling pathways can lead to many adverse effects, it is essential to develop efficient approaches to assess TH system disruption by various stressors. The initial step in TH synthesis is the uptake of iodide anions (I^−^) from the bloodstream into thyroid follicular cells via sodium/iodide symporter (NIS). The proper function of NIS is critical for iodide transport and thyroid hormone synthesis. This membrane glycoprotein, coded by the SLC5A5 gene (Martín et al. 2019), is located at the basolateral part of thyrocytes. Under physiological conditions, NIS uses an influx of Na^+^ to symport I^−^ against its gradient, achieving intracellular levels 20 to 40 times higher than those in the bloodstream (Portulano et al. 2014). Other monovalent ions with a similar ionic radius, such as the xenobiotic perchlorate and the naturally occurring compound thiocyanate in their ionic state, can also be transported and, therefore, can competitively inhibit iodide uptake (Jones et al. 1996). Inhibition of NIS-mediated iodide uptake impairs TH synthesis, which is associated with decreased TH concentration and hypothyroidism. The role of NIS has also been implicated in thyroid and other cancers (Micali et al. 2014). Importantly, functional NIS plays a central role in the use of radioiodine therapy in thyroid cancer. NIS transported I^−^ radioisotopes are used to diagnose, treat, and monitor thyroid pathologies (Chung et al. 2018; Ravera et al. 2017), and NIS can also be used as a diagnostic transgene (Kitzberger et al. 2022; Spitzweg et al. 2021). Thus, compounds interfering with NIS could affect not only cancer development but also the sensitivity of its diagnosis and its treatment efficiency by radioactive iodide.

Given the economic constraints of toxicologically assessing the plethora of potentially adverse compounds within the exposome scenario, ethical considerations on animal testing, and cross-species extrapolation issues (Caloni et al. 2022), modern toxicology can no longer solely rely on classical in vivo testing. Development and implementation of new approach methodologies are urgently needed for regulatory purposes and prioritization of the THSD chemicals. Methodological scarcity is evident, especially in the context of THSD and NIS impairment assessment, with limited knowledge on the comparability of different assays. Moreover, the few available in vitro systems lack biotransformation capacities. These limitations need to be addressed and rectified within the foreseeable future.

Historically, the iodide uptake capacity of the thyroid gland was studied using radiometric detection of the ^125^I isotope (Pavelka 2015). However, the need for broader method applicability in labs not equipped to handle radioactive materials has led to the development of a less demanding non-radioactive detection method. In this alternative approach, iodide levels can be detected spectrophotometrically using the Sandell–Kolthoff reaction (SK), which is based on the catalytic effect of iodide on the reaction between cerium (IV) and arsenic (III) (Waltz et al. 2010). In vitro, iodide uptake can be monitored in cell models expressing NIS, such as primary thyroid cells (Hingorani 2010; Ravera et al. 2017) and rat thyroid-derived FRTL-5 cells (Buckalew et al. 2020), which express NIS endogenously. Transgenic in vitro models overexpressing NIS, based on, e.g., HEK293T cells (EPAhNIS) (Hallinger et al. 2017; Wang et al. 2019), human thyroid carcinoma cells TPC-1, and 8505C cells (Read et al. 2022) can provide a suitable option for high-throughput testing. Previously, these cell models were utilized jointly with the radiometric isotope detection assay to investigate iodide uptake. More recently, it has been shown that the SK reaction could be utilized as a possible alternative (Dong et al. 2019) together with some in vitro systems. Still, the applications of this setup have been limited so far. There is a lack of information on the consistency or potential differences of the results obtained using various in vitro models. Moreover, in vitro models often fail to capture the inherent complexity of organ systems, particularly regarding the metabolic capacities for the (in)activation of chemicals. As many endocrine-disrupting chemicals are expected to be altered by the xenobiotic metabolism machinery, metabolically non-capacitated in vitro test systems fall short in terms of regulatory relevance. These limitations have been termed as the “bottleneck” of in vitro toxicological test development (Coecke et al. 2006).

Tackling the above-stated issues, this study aims to characterize a set of in vitro models and their suitability and sensitivity for NIS-inhibition assessment as well as comparability of results across various in vitro models and different assay formats. It first aims to assess the compatibility of the employed cellular test systems with the SK-related spectrophotometric approach and readout. Therefore, we investigated NIS gene and protein expression and activity measured by SK reaction in wild-type and established transgenic cell lines. It also investigates the inter-assay and cross-species comparability of the NIS-inhibition results by parallel application of human cell-based and rat cell-based models and assesses their sensitivity and reproducibility. We also utilized the US EPA in silico tool “SeqAPASS” to compare cross-species conservation of potential chemical-to-protein interactions.

Second, to overcome biotransformation limitation and increase physiological relevance, the study aimed to establish a methodology for augmenting the NIS-inhibition assay with an external biotransformation system (BTS). An important goal was the optimization of the NIS-inhibition assay compatibility with BTS to enable the assessment of the impact of metabolization on the NIS inhibitory effects of the model chemicals.

## Materials and methods

### Chemicals

The performance of the NIS-inhibition assay was characterized using different in vitro models (see respective sections below) and a set of 23 selected compounds. These chemicals represent diverse human exposure-relevant chemical groups such as pharmaceuticals, industry chemicals, consumer products, pesticides, and perfluorinated compounds (Table 1, Figure S1). 21 of these chemicals were prioritized as potential THSD compounds and reference controls by collaborative effort within the EU Horizon 2020 project “ERGO” (Endocrine Regulation of Growth and Development), which aimed to establish a network of adverse outcome pathways for TH disruption across different vertebrates, enhance biomarkers and improve test methods to more accurately predict human health impacts (Holbech et al. 2020). Two additional chemicals, methoxychlor and etoxazole, described as NIS inhibitors previously (Wang et al. 2018), were added to the set to enable the assessment of the performance of the BTS pretreatment step for compounds with high octanol–water partition coefficient (Kow) (Table S1). Further details on the chemical selection criteria are provided in the Supplementary Information (SI, section Material and Methods).

**Table 1:** Effect of the tested chemicals in NIS-inhibition assay on HEK293T NIS02, HEK293T NIS C, and FRTL-5 cell lines expressed as IC_50_ values (µM; mean ± SD; mean from ≥ 3 independent experiments). *—IC_50_ values in the cytotoxic concentration range are shown in italics; †—IC_50_ levels, which were not in the cytotoxic concentration range, but cytotoxicity occurred at greater exposure levels; n.e. no statistically significant effect. *PCPs* Personal Care and Consumer Products

Abbrv	Chemicals	Chemical category	Highest test. conc (µM)	HEK293T NIS 02	HEK293T NIS C	FRTL-5
AMP	Ampicillin	Antibiotics and antimicrobials	100	n.e	n.e	n.e
BP2	2,2′-4,4′-Tetrahydroxy Benzophenone	PCPs	100	n.e	n.e	n.e
BPA	Bisphenol A	Industrial chemical	500	111 ± 75	*309* ± 2.5*	*302* ± 197*
CBZ	Carbamazepine	Pharmaceuticals	100	n.e	n.e	n.e
DBP	Dibutylphthalate	Industrial chemicals	100	51.7 ± 23.1	n.e	*92.3* ± 11.8*
DON	Deoxynivalenol	Mycotoxins	100	n.e	n.e	n.e
ETU	Ethylene thiourea	Industrial chemical	100	n.e	n.e	n.e
ETX	Etoxazole	Insecticide	100	7.1 ± 4.3	11.7 ± 8.0†	8.05 ± 1.2
IOP	Iopanoic acid	Pharmaceuticals	100	n.e	n.e	n.e
MET	Methoxychlor	Pesticide	500	11 ± 8.7	n.e	218 ± 4.3
MMI	Methimazole	Pharmaceuticals	100	n.e	n.e	n.e
PCL	Perchlorate	Industrial chemicals	100	1.65 ± 0.57	14.9 ± 8.3	5.1 ± 4.6
PFOA	Perfluorooctanoic acid	Perfluorinated chemicals	100	n.e	n.e	n.e
PFOS	Perfluorooctane sulfonate	Perfluorinated chemicals	100	30.9 ± 21.7	n.e	9.3 ± 4.3
PTU	6-Propylthiouracil	Pharmaceuticals	100	n.e	n.e	n.e
RSC	Resorcinol	Industrial chemicals	10,000	4057 ± 2048	n.e	4709.5 ± 562
SA	Salicylic acid	PCPs	100	n.e	n.e	n.e
SMX	Sulfamethoxazole	Antibiotics and Antimicrobials	100	n.e	n.e	n.e
T3	3,3′,5-Triiodo-L-thyronine	Thyroid hormone	100	n.e	n.e	n.e
T4	3,3′,5,5″-Tetraiodo-L-thyronine	Thyroid hormone	100	n.e	n.e	n.e
TBBPA	Tetrabromobisphenol A	Industrial chemicals	100	*30.1* ± 11.8*	*54* ± 27.1*	*91.8* ± 10.8*
TCS	Triclosan	Antibiotics and antimicrobials	100	18.5 ± 9.9†	*34.8* ± 23.5*	*39.3* ± 18.9*
TPP	Triphenyl phosphate	Industrial chemicals	100	42.8 ± 18.9	n.e	92.6 ± 24.1

### Cell models

Several cell lines have been used in the study. FRTL-5, a spontaneously immortalized rat thyroid follicular cell line with the intrinsic expression of NIS; Nthy-ori 3–1, an immortalized human thyroid follicular epithelial cell line; HEK293T, immortalized human embryonic kidney cells, either wild-type or transfected with human NIS gene. For the preparation of the transfected HEK293T cell models, a human NIS sequence from the hSLC5A5 gene was retrieved from NCBI (NM_000453.3). This sequence was used as a template for constructing a lentiviral vector, created by the service provider Vector Builder (USA). The resulting vector, designated VB200915-1039wfn, contained the gene of interest (hSLC5A5) under the transcriptional control of the CMV promoter (Figure S2). In addition, to enable the selection of the transfected cells, an EGFP gene was linked with a puromycin resistance gene, both under the control of a CMV promoter (pLV[Exp]-EGFP:T2A > hSLC5A5[NM_000453.3]). For further details and more information on the cultivation conditions and characterization of the employed cell lines, which included the assessment of NIS gene and protein expression and their iodine uptake, see the SI.

### NIS-inhibition detection

The NIS-inhibition assay corresponded to the method previously described by Dong et al. (2019) with minor modifications. Cells were seeded in poly-L-lysine-coated (Sigma-Aldrich) 96-well plates (TPP, Switzerland) at 15,000 cells/well concentration in 200 µL of culture medium. Cells were grown for 48 h prior to exposure, then the media was removed, and the cells were washed twice with Hank’s balanced salt solution (HBSS, Roth).

The cells were exposed to a dilution series of the test compounds for 2 h at 37 °C in 100 µL of uptake buffer (HBSS, 5 µM NaI, 0.5% v/v MeOH). The concentration range for most of the tested chemicals was 0.78 to 100 µM (Tables 1 and S2). In the case of BPA and MET that repeatedly elicited low but statistically significant NIS-inhibition effect at 100 µM, the exposure concentration range was extended to 500 µM, to obtain better covered concentration–response curves. In the case of resorcinol, which has several orders of magnitude higher water solubility than most of the other chemicals (Table S1), the concentration range was set to 80–10,000 µM, because its testing was harmonized with a cross-species in vivo study design (Van Dingenen et al. 2024). The final concentration of solvent (MeOH) in the assay was 0.5% in the plate wells with the tested chemicals as well as in the appropriate control wells (cell-free background control—wells w/o cells with the uptake buffer; positive control—wells w/cells exposed to the uptake buffer; negative control—cells exposed to the HBSS w/o NaI).

After a 2-h exposure in a shaker incubator (Thermoshaker, Biosan) at 37 °C and 120 rpm, all wells were carefully washed with HBSS to remove the iodide not absorbed by the cells. A calibration series of NaI was run on each plate to allow for the quantification of iodide levels absorbed by the cells. The iodide level content in the plates was sensitively detected using the SK reaction, where cells were lysed with arsenic solution (25 mM NaAsO_4_, 0.5 M H_2_SO_4_, 0.2 M NaCl; Sigma-Aldrich) and ceric solution (25 mM (NH_4_)_4_Ce(SO_4_)_4_, 0.5 M H_2_SO_4_; Sigma-Aldrich). Absorbance was measured after 20-min incubation using a plate reader (Biotek Synergy MX) at 415 nm. The incubation time was selected based on the literature (Dong et al. 2019) and pilot optimization experiments. The cell exposure duration for 1, 2, 4, and 6 h was tested with 2-h exposure providing the best sensitivity-variability ratio (data not shown). In parallel with the NIS-inhibition assay, the effect of the same concentrations of the chemicals on the cell viability was always assessed using a Neutral red uptake assay, and the results were orthogonally confirmed by the CelltiterGlo assay (Promega). The inactive chemicals were assessed in 3 independent experiments on both HEK293T NIS 02 and HEK293T NIS C cells, and in 2 experiments on FRTL-5 cells. Active chemicals were assessed in 3 experiments on FRTL-5 cells and ≥ 3 experiments on HEK293T NIS 02 and HEK293T NIS C cells, respectively. For more details on the methodology of the NIS-inhibition assay and the cellular viability assessment, see the SI.

### NIS biotransformation augmentation

We augmented the NIS-inhibition assay with an external BTS (consisting of benzoflavone/phenobarbital-induced, male rat-derived post-mitochondrial supernatant, S9 fractions (Moltox, USA), and respectively, added cofactors, as described further below) to enhance the physiological relevance of the in vitro assay and assess if biotransformation would affect the NIS inhibiting capacities of the tested chemicals. Information on the BTS characterization, as provided by the producer, is given in the SI section “Biotransformation” together with the in silico biotransformation prediction procedure conducted by the BioTransformer 3.0 tool. BTS-driven metabolization was conducted as a preincubation step of the tested chemicals before the NIS assay, with the three following exposure variants: (1) fully active BTS (labeled “w/S9, w/cof.”), including all cofactors and the active S9 fraction to assess metabolic (in)activation. (2) Heat-inactivated BTS (“D S9, w/cof.”), as the main BTS-relevant control, including all cofactors and heat-inactivated (denatured) S9 to assess the potential impact of the proteins present within the BTS on chemical bioavailability. S9 fractions were denatured by heating a separate aliquot for 10 min at 95 °C on a heating coil. (3) Control without BTS (labeled “w/o S9, w/cof.”) included all cofactors without S9 fractions to account for the effect of cofactors alone on the cells. The preincubation of test chemicals with S9 fraction and the cofactors was performed in amber glass vials for 5 h at 37 °C in the dark. BTS reaction mixtures were prepared in HBSS, including 0.5% (v/v) MeOH and twofold increased (2X) highest non-cytotoxic concentrations of test chemicals causing NIS inhibition (see paragraph further below). In addition, BTS reaction mixtures contained the final reaction concentrations of 0.01 mg/mL S9 and 5 mM MgCl_2_. Moreover, phase 1 and 2 biotransformation-relevant cofactors with the final reaction concentrations of 200 µM beta-nicotinamide adenine dinucleotide phosphate reduced tetrasodium salt (NADPH, Thermo Scientific™), 500 µM uridine 5′-diphosphoglucuronic acid trisodium salt (UDPGA, Sigma-Aldrich), 2 µM adenosine 3′-phosphate 5′-phosphosulfate triethylammonium salt (PAPS, Sigma-Aldrich), and 200 µM L-Glutathione (GSH, Thermo Scientific™) were also included in the BTS reaction mix. For further details on stock preparation and dilution steps pertinent to the BTS, please consult the SI section “Biotransformation”.

After the 5-h preincubation with the BTS, the BTS reaction mixture was mixed 1:1 with the NIS assay buffer with two-time elevated NaI concentration (HBSS, 0.5% MeOH and 10 µM NaI) to dilute down the BTS components not to interfere with the NIS-inhibition assay and introduce NaI to the solution. These prepared mixtures were used to prepare the exposure dilution series of the exposure variants (“w/S9, w/cof.”, “D S9, w/cof.”, “w/o S9, w/cof.”) by mixing with the NIS assay buffer. These three dilution series sets were then used for the standard NIS assays. Each compound was also tested without any preincubation in parallel on the same plate (defined as “standard method” (SM) in the results). The NIS-inhibition assay for the four exposure variants was carried out in a standard way as described above (for more details see SI).

The BTS-augmented assay included the same tested concentration ranges of the chemicals showing NIS inhibition at the non-cytotoxic concentrations as in the previously described standard NIS assay, except for Triclosan, since its 100 µM concentration was cytotoxic according to Neutral red uptake assay (NR) and CellTiter-Glo Luminescent Cell Viability Assay (CTG) results in the standard NIS assay. Hence, it was decided to use a lower concentration range (0.24 to 30 µM) in the BTS-augmented experiments. Further details on the BTS description are provided in the SI, together with the in silico biotransformation prediction procedure.

### Data analysis

For the NIS-inhibition assay and biotransformation-augmented NIS assay, raw data were acquired from a spectrophotometer. The raw data from the row containing iodide calibration was used to calculate iodide uptake levels using an iodide calibration curve via Hill non-linear regression curve fitting (inhibitor vs. response, 4 parametric variable slopes; GraphPad Prism (GraphPad Software, San Diego, USA)). To calculate percent inhibition, the data were normalized to the background and positive controls (min-to-max normalization). The cell-free background control served as a proxy for cells with no iodide uptake, i.e., completely inhibited or not expressing NIS, corresponding to the non-transfected HEK293T cells, as shown in the Results section. Opposingly, the positive control consisted of cells with uninhibited NIS activity, providing maximum potential iodide uptake. The normalized data were used to generate the concentration–response curves in GraphPad Prism. A non-linear regression curve was fitted to the data using a 3-parametric Hill equation to determine the IC_50_ (the concentration of the inhibitor that reduces iodide uptake by 50%) or IC_20_ (concentration of the inhibitor that reduces the iodide uptake by 20%) values for each compound and assay variant. For the data obtained from the biotransformation-augmented NIS assay, a two-way ANOVA (mixed model, accounting for the factors concentration, BTS setup, and experimental replication) was used to analyze the statistical significance of effect differences among various biotransformation setups (“w/S9, w/cof.”, “D S9, w/cof.”, “w/o S9, w/cof.”, and “SM”; alpha level = 0.05). ANOVA test criteria (normality of residuals and homoscedasticity) were assessed (Spearman’s test for homogeneity of variance, Shapiro–Wilk and Kolmogorov–Smirnov tests for normality of residuals, visual inspection of QQ-plots and residual plots) and met, respectively.

The data from NR and CTG viability assays were normalized to the positive control response, representing 100% viable cells. The normalized data were plotted using GraphPad, facilitating the calculation of IC_20_ values (the concentration of the inhibitor where the response is reduced by 20%) using non-linear regression, as stated above. The concentrations of chemicals that caused a 20% or more reduction in viability were considered cytotoxic.

### Cross-species comparison of NIS protein

To assess the potential relevance of NIS inhibition observed with in vitro models based on human and rat cells for other species, we utilized the Sequence Alignment to Predict Across Species Susceptibility (SeqAPASS) tool developed by the US EPA (https://seqapass.epa.gov/seqapass/). For further details, see the SI.

## Results and discussion

Efficient approaches enabling the identification and prioritization of potential thyroid hormone-system-disrupting chemicals (THSDCs) acting through various molecular initiating events (MIEs), which are related to disrupted thyroid hormone homeostasis, are urgently needed (Haigis et al. 2023; Vergauwen et al. 2024). Inhibition of iodide uptake in thyrocytes mediated by NIS is one of the crucial identified MIEs, which can be affected by a diverse set of THSDCs (Friedman et al. 2016; Hallinger et al. 2017; Hornung et al. 2018; Olker et al. 2019; Titus et al. 2008; Wang et al. 2019, 2018). The effect of THSDCs on NIS activity has been connected with the decrease of TH production in the thyroid gland and significant health effects, namely during development (De Groef et al. 2006; Zimmermann 2011). Importantly, as NIS is often used as an important theragnostic gene in radionuclide uptake-based carcinoma treatment (Chung et al. 2018; Kitzberger et al. 2022; Spitzweg et al. 2021), NIS inhibition by xenobiotics could also adversely affect diagnostic and treatment methods and potentially jeopardize therapy outcomes since NIS activity in carcinoma cells is being inversely connected with the recurrence and metastatic potential of the thyroid carcinoma (Oh and Ahn 2021).

This study brings important information on NIS assay performance across different in vitro models and advances its in vivo relevance and applicability by developing biotransformation-competent assay set up for NIS-inhibition screening purposes by incorporating BTS capacities into the assay. The most crucial results are outlined and discussed in the following sections, with additional material given in the SI.

### NIS gene and protein expression in candidate cell lines

We examined SLC5A5 (NIS) gene expression in ten model cell lines. While human thyrocytes-derived cell line Nthy-ori 3–1, together with the commonly used human cell line HEK293T, were without detectable NIS expression, the transfected HEK293T cell lines, together with the rat FRTL-5 cell line, had significant NIS gene expression levels (Figure S3), as compared to commercially available human and rat total thyroid RNA samples. The highest gene expression was detected in the cell cultures HEK293T NIS 01 and 02 as well as with single-cell clone line HEK293T NIS C. Among these, HEK293T NIS C showed the highest NIS gene expression, followed by HEK293T NIS 02 and HEK293T NIS 01, while the rest of the transfected cell lines were removed from further evaluation due to low NIS gene expression and in some cases also poor growth characteristics (Figure S3).

Western blot analysis revealed protein bands corresponding to NIS in the prioritized transfected cell lines, confirming the presence of NIS protein. No NIS protein expression was observed in Nthy-ori 3–1 or non-transfected HEK293T cell lines (Figure S4). The three transfected human cell lines with good stable NIS protein expression also across higher cell culture passages (HEK293T NIS C, NIS 01, and NIS 02), along with the endogenously NIS-expressing rat FRTL-5 cell line, were selected for further characterization of NIS activity and their suitability for NIS-inhibition measurement.

### Effects on NIS activity

The NIS gene and protein expression characterized via qPCR and Western Blot analysis (Figure S3 and S4) corresponded to NIS-mediated iodide uptake assessed by SK reaction. There was no iodide uptake due to the absence of NIS protein in the HEK293T and Nthy-ori 3–1 cell lines, where the recorded responses (absorbance at 415 nm) corresponded to the cell-free controls (Figure S5B). However, even though the NIS gene and protein expression patterns were similar between the HEK293T NIS 01 and NIS 02 cell lines (Figure S4), the former depicted lower iodide uptake in the pilot experiments (Figure S5B) and was excluded from further investigations.

NIS-expressing cell lines HEK293T NIS 02, HEK293T NIS C, and FRTL-5 with good iodide uptake, were finally selected for NIS-inhibition assessment by chemical exposure. The two HEK293T-derived models were employed for the detailed characterization to verify if they show the same performance characteristics as they share the same NIS-expression gene construct. The characterization was performed along with careful evaluation of cellular viability, which is crucial in the NIS-inhibition assay, as cytotoxic effects could be mistakenly interpreted as NIS inhibition, leading to false-positive results.

**Fig. 1:**
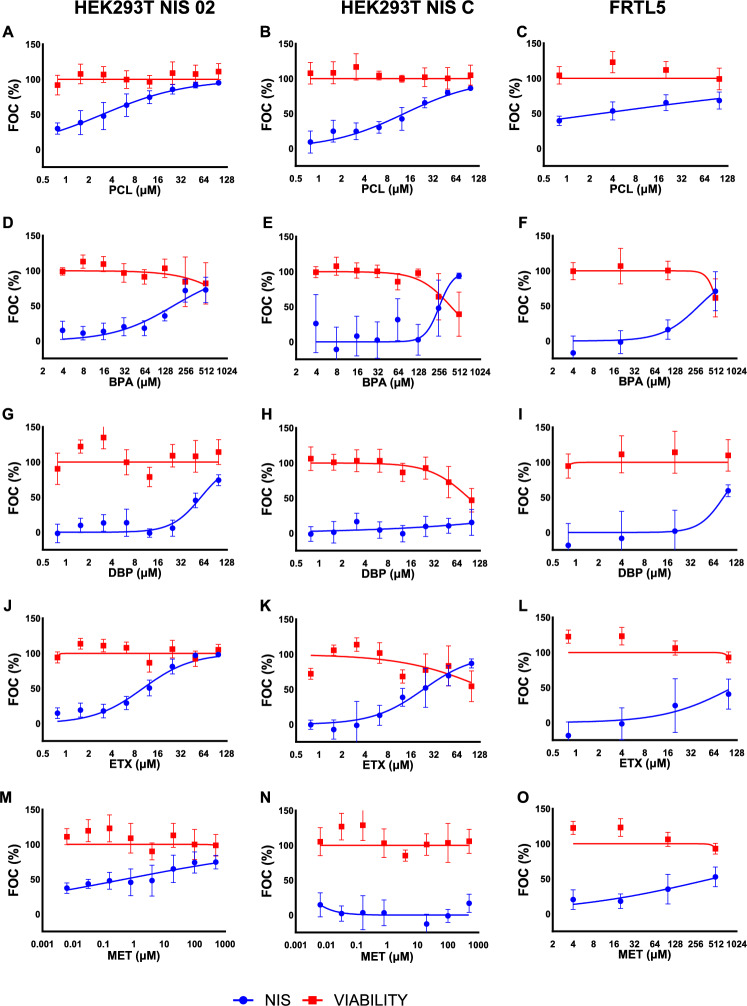
Detected effects of the first part of tested compounds on NIS inhibition (blue curve) and cell viability (red curve) in two hNIS transfected cell lines, namely HEK293T NIS02 (left), HEK293T NIS C (middle), and rat thyroid cell line FRTL-5 (right). Points represent the total mean from at least three independent experiments (*n* ≥ 3 experimental units, *N* ≥ 9 observational units/technical replicates) per tested concentration, with whiskers representing ± SD of all observations. The data are shown as FOC%, indicating the fraction of control. For NIS inhibition, the cell-free control (no iodide uptake) serves as a reference, with an increase in FOC% reflecting enhanced inhibition of iodide uptake. For cell viability, a decrease in FOC% indicates reduced viability relative to the NaI control (100% viable cells). The names and abbreviations of the tested chemicals and their respective IC_50_ NIS-inhibition values are provided in Table 1, the cytotoxicity IC_20_ values are in Table S2. The tested concentrations ranged from 0.78 to 100 µM, except for BPA and MET, which were tested up to 500 µM (Colour figure online)

**Fig. 2:**
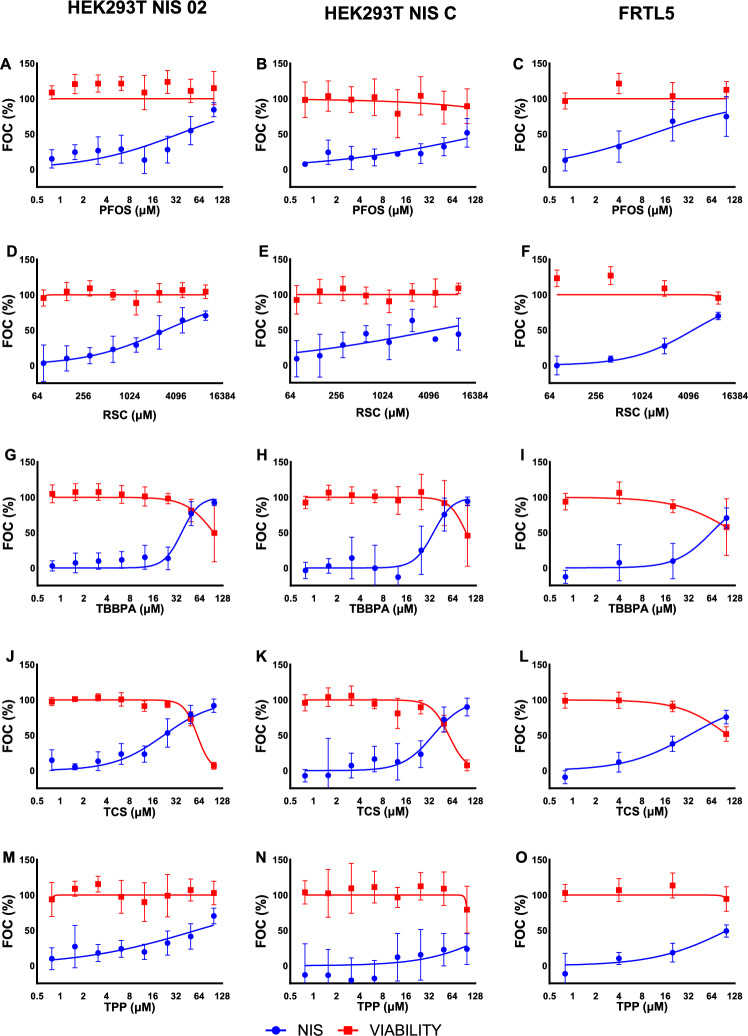
Detected effects of the second part of tested compounds on NIS inhibition (blue curve) and cell viability (red curve) in two hNIS transfected cell lines, namely HEK293T NIS02 (left), HEK293T NIS C (middle), and rat thyroid cell line FRTL-5 (right). Points represent the total mean from at least three independent experiments (*n* ≥ 3 experimental units, *N* ≥ 9 observational units/technical replicates) per tested concentration, with whiskers representing ± SD of all observations. The data are shown as FOC%, indicating the fraction of control. For NIS inhibition, the cell-free control (no iodide uptake) serves as a reference, with an increase in FOC% reflecting enhanced inhibition of iodide uptake. For cell viability, a decrease in FOC% indicates reduced viability relative to the NaI control (100% viable cells). The names and abbreviations of the tested chemicals and their respective IC_50_ NIS-inhibition values are provided in Table 1, the cytotoxicity IC_20_ values are in Table S2. The tested concentrations ranged from 0.78 to 100 µM, except for RSC, whose maximal tested concentration was 10,000 µM (Colour figure online)

The current study applied a non-radioactive approach (SK reaction) to measure NIS-mediated iodide uptake inhibition by 23 model chemicals from diverse use categories (Table 1, Figs. 1 and 2). Four of them (PCL, TCS, BPA, DBP) have been previously tested using the same method on other in vitro models (Dong et al. 2019; Waltz et al. 2010; Wu et al. 2016; for more details see Table S2). Nevertheless, there are data for 16 of the chemicals from radioactivity-based method (RAIU) on several in vitro models (see Table S2), which enables the assessment of comparability of the results across the two different assay formats and the various in vitro models. RAIU detection was employed on hNIS-HEK293T-EPA cell model in the current version of ToxCast (US EPA 2024), papers building on data from the earlier ToxCast datasets (Wang et al. 2019, 2018), studies using the ToxCast cell model (Buckalew et al. 2020; Hallinger et al. 2017), FRTL-5 cell model (Buckalew et al. 2020; Waltz et al. 2010), or HeLa cells transfected with NIS genes from different vertebrates (Concilio et al. 2020). Table S2 in Supplementary Information provides a detailed overview of the results from NIS inhibition and cytotoxicity in the three in vitro models used in our study along with the relevant data across the studies in literature and the ToxCast database.

The results of our study, where all chemicals were assessed in parallel on three different in vitro models by the same SK reaction-based method, demonstrate some differences in the sensitivity of the used in vitro models, regarding both NIS inhibition and cytotoxicity (Table 1). To provide transparent results, we report not only the mean effective values but also their standard deviations across at least 3 independent experiments for both endpoints (Table S2), which are mostly not reported in previous literature. Nine of the tested chemicals were detected as active NIS inhibitors with six of them active at non-cytotoxic concentrations in at least two of the three in vitro models used in our study (HEK293T NIS02 and FRTL-5; Table 1, Figs. 1 and 2). For eight of these chemicals, our data are consistent with the literature (Table S2). In the case of RSC, we have detected NIS inhibition at concentrations beyond the range tested in ToxCast. The lower concentrations corresponding to those tested in ToxCast, where it was categorized as inactive, also did not cause significant inhibition in our assays. TPP was active in two of our in vitro models without significant cytotoxicity, allowing us to quantify its IC_50_ (Fig. 2, Table 1, S2). It was also recognized as active in ToxCast, but there was also significant cytotoxicity at the same concentrations (Table S2). For TBBPA, NIS-inhibition activity was very close to the cytotoxicity effect in both our study and ToxCast, so it cannot be classified as a specific NIS inhibitor.

Our data also align with the literature for the five inactive chemicals for which any previous data were available. Only in the case of PFOA, recognized as inactive in all three cell models in our study, the literature data were ambiguous. It was also recognized as inactive in ToxCast (US EPA 2024) and Wang et al. (2018), which was based on an older version of ToxCast dataset, but it was active at relatively high concentrations in RAIU in FRTL-5 cells and the hNIS-HEK293T-EPA cell model in Buckalew et al. (2020). Since we used the same rat cell model and did not detect NIS inhibition, it might be possible that the RAIU assay was more sensitive than the SK assay. However, this is not supported by the ToxCast data (US EPA 2024), which do not show significant NIS inhibition with the same detection method and human cell model as in Buckalew et al. (2020). Thus, considering this is the only discrepancy in categorization of chemicals between our study and the literature, the SK assay appears to have similar sensitivity to the more complex RAIU assay. The IC_50_ levels detected using HEK293T NIS02 and FRTL-5 cell models were also very similar to those detected with the RAIU method in the literature (Table S2).

There are differences in cytotoxic concentrations of BPA, TCS, and PFOS across various in vitro models across studies. In our study and literature, BPA showed NIS inhibitory effects at relatively high µM IC_50_ concentrations near cytotoxicity limits. PFOS and TCS demonstrated NIS inhibition with lower IC_50_ values, and cytotoxicity findings varying across studies (Table S2). For an independent orthogonal cytotoxicity assessment, we have evaluated their cytotoxicity also using the CTG assay, which confirmed identical cytotoxicity cutoff values in both the NR and CTG assays (Figure S6). We attribute discrepancies primarily to variations in cell sensitivity across the studies but differences in definition and calculation of cytotoxicity cutoff values can play a role here. These findings emphasize the need to assess cytotoxicity alongside NIS inhibition, ensuring that identified NIS inhibitors are genuine and not merely artifacts of cell death, which can obscure inhibition effects. This has been also highlighted throughout the application of the Radioactive Iodine Uptake assay (RAIU) in a study using hNIS-HEK293T-EPA test model, which emphasized the importance of distinguishing between true NIS inhibition and nonspecific reductions in iodide uptake caused by cell death or compromised cellular health (Buckalew et al. 2020).

In our study, the HEK293T NIS 02 model showed the highest sensitivity to NIS inhibition among the three tested models, while NIS C was more prone to cytotoxic effects and exhibited lower sensitivity to NIS inhibition, despite having higher NIS-expression levels (Figure S3, S4; Table 1). The increased cytotoxicity in the NIS C model may result from the specific genomic integration site of the NIS-expression construct, potentially affecting genes vital for cellular metabolism. Alternatively, high NIS activity in this model might disrupt cellular homeostasis. Comparisons among HEK293T NIS 01, NIS 02, and NIS C clones—all transfected with the same vector—highlight that assay performance depends on factors beyond the vector itself. The number and genomic location of integrated gene constructs may influence the expression of other essential genes and possibly also the translocation of NIS protein to the membrane (Oh and Ahn 2021). These factors could indirectly impact cell model performance. This should be taken into account when comparing the data from different in vitro models overexpressing NIS. This can at least partly account for the differences in IC_50_ values across various human NIS-based in vitro models.

The rat FRTL-5 model, with intrinsic NIS expression, produced NIS-inhibition data generally consistent with the NIS 02 cell model (Figs. 1, 2, and Table 1), identifying the same compounds as active or inactive with comparable or slightly higher IC_50_ values. This consistency supports the relevance of the newly established human NIS 02 cell model, which makes it suitable for NIS-inhibition studies. While the FRTL-5 cell line showed comparable results as HEK293T NIS02, it was not as suitable model for efficient testing of chemicals due to its slow growth rate and expensive growth medium. In addition, NIS C with its lower sensitivity to NIS inhibition and higher sensitivity to cytotoxicity is less suitable for this purpose.

Although differences between our results and published data were generally minor, our results show there can be sensitivity differences even among similar in vitro models expressing the same NIS gene, as seen between HEK293T NIS02 and NIS C cell lines, which share the same transfection vector. While various in vitro models can effectively identify stronger NIS inhibitors, their ability to identify weak inhibitors may be compromised if they are more sensitive to general cytotoxic effects. IC_50_ values for NIS inhibition should be interpreted cautiously in parallel with cytotoxic effects and ideally validated with models of greater in vivo relevance. A key difference from in vivo systems remains the absence of biotransformation mechanisms in these in vitro models.

### Effect of biotransformation on modulating NIS-mediated iodide uptake

**Fig. 3:**
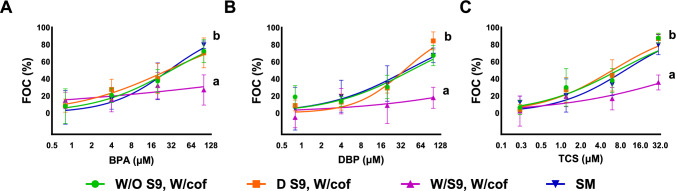
Results from the biotransformation (BTS)-augmented NIS-inhibition assay using HEK293T NIS02 cells under four experimental conditions (detailed below), each assessed in 3 independent experiments. The Y-axis shows FOC% (fraction of no uptake control), representing NIS inhibition, where 100% FOC indicates complete inhibition (based on cell-free controls). The names and abbreviations of the tested chemicals are provided in Table 1. Dots represent the mean normalized NIS activity per exposure concentration, whereas the whiskers represent the standard deviation across replicates. The statistically significant differences among BTS setups are indicated by different letters (*p* < 0.05). The BTS setups were as follows: W/O S9 and W/cof (green curve), representing chemicals treated without the S9 fraction but with all cofactors added; D S9, W/cof (orange curve), where chemicals were treated with denatured S9 and all cofactors added; W/S9, W/cof (purple curve), indicating treatment with both S9 fraction and cofactors; and SM (blue curve), the standard NIS-inhibition assay method (Colour figure online)

**Fig. 4:**
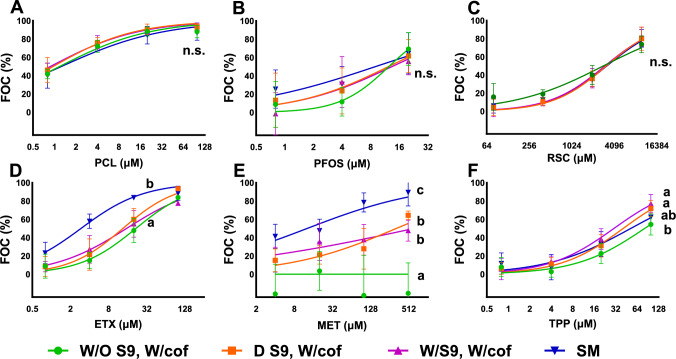
Results from the biotransformation (BTS)-augmented NIS-inhibition assay using HEK293T NIS02 cells under four experimental conditions (detailed below), each assessed in 3 independent experiments. The Y-axis shows FOC% (fraction of control), representing NIS inhibition, where 100% FOC indicates complete inhibition (based on cell-free/no uptake controls). Chemical abbreviations are explained in Table 1 and Table S1. Dots represent the mean normalized NIS activity per exposure concentration, whereas the whiskers represent the standard deviation across replicates. The BTS setups were as follows: W/O S9 and W/cof (green curve), representing chemicals treated without the S9 fraction but with all cofactors added; D S9, W/cof (orange curve), where chemicals were treated with denatured S9 and all cofactors added; W/S9, W/cof (purple curve), indicating treatment with both S9 fraction and cofactors; and SM (blue curve), the standard NIS-inhibition assay method. The statistically significant differences among BTS setups are indicated by different letters (*p* < 0.05), “n.s.” indicates no significant differences (Colour figure online)

To better mimic the in vivo situation, the established NIS-inhibition assay was retrofitted with an external biotransformation system (BTS). Based on its demonstrated sensitivity and applicability as well as human relevance, the HEK293T NIS 02 cell line was selected as the test model for investigating the effect of BTS on the overall assay outcomes. Biotransformation was addressed by incorporating a pre-exposure phase, which included the incubation of the test chemicals with S9 and respective cofactors. A set of compounds previously identified (Table 1, Figs. 1 and 2) as effective NIS inhibitors in HEK293T NIS 02 cells at non-cytotoxic concentrations, namely BPA, DBP, ETX, MET, PCL, PFOS, RSC, TCS, and TPP, was chosen for these follow-up experiments. Both NIS inhibition and cell viability were assessed concurrently (Figs. 3, 4, S7).

Incorporating BTS into the NIS assays impacted the effects of the tested compounds in three different ways, as compared to the standard NIS assay. First, we recorded a statistically significant reduction in NIS inhibition for DBP, TCS, and BPA (Fig. 3) in the subgroups utilizing a fully active BTS (“W/S9, W/cof”). Contrarily, the variants consisting of denatured S9 (“D S9, W/ cof”) and cofactors-only (“W/O S9, W/cof”) did not lead to a statistically significant impact on NIS inhibition, also when compared to the NIS method without BTS (“SM” – standard method) that was run in parallel.

We derived IC_20_ values and their ratios for all NIS inhibitors, that depicted statistically significant differences between fully active BTS systems and respective controls for a facilitated quantitative comparison by dividing the IC_20_ of the fully active BTS setup (“W/S9, W/cof”) by the IC_20_ of the denatured BTS setup (“D S9, W/cof”) (see Table S3 for the summary). IC_20_ values were prioritized for this comparison as the NIS inhibition often did not reach up to 50% effect in the fully active BTS setup (Fig. 3). The preincubation in fully active BTS setup increased IC_20_ values by approximately 5 (TCS), 6.3 (BPA), and more than ninefold (DBP) as compared to the denatured BTS setup (Table S3). According to those results, these compounds were metabolized by the added BTS and subsequently caused less NIS inhibition. The impact of compound bioavailability can be ruled out as the control setups showed no statistically significant differences in IC_20_ values among the standard method and the different BTS control variants.

Second, for another group of investigated compounds (PCL, PFOS, and RSC; Fig. 4A, B, C), no significant differences were observed among the effects of the variants with the active BTS setup and BTS-related control setups. As the concentration–response patterns were almost identical in between test setups, we deduced that these respective compounds are not impacted by biotransformation, at least in their NIS-inhibition ability within this specific in vitro assay.

Finally, a set of compounds (ETX, MET and TPP, Fig. 4D, E, F) demonstrated varying, setup-specific concentration–response patterns, which can most likely be attributed to kinetic compartmentalization effects, impacting bioavailability. Importantly, no biotransformation-related effect changes were detected for these compounds, as there were no statistically significant differences between their effects in fully active BTS setups compared to setups containing denatured S9. As mentioned previously, ETX and MET were specifically added to the set of test compounds due to their hydrophobicity (log K_ow_ > 5). Indeed, differences in effect measures are evident for ETX (log K_ow_ = 5.6) between the standard method (“SM”) and all BTS setups that include a 5-h preincubation step (Fig. 4D and Table S3). Although glass vessels were utilized, preincubation likely diminished free available ETX concentrations by binding to the vessel or losses of the chemical by evaporation, although the incubation vials were tightly sealed and with minimal head space. Interestingly, for MET (log K_ow_ = 5.1), effect measures differed similarly between the standard method and all BTS-related setups; however, a statistical difference was also computed between S9-bearing setups (“W/S9, W/cof” and “D S9, W/cof”) and the “W/O S9, W/cof” control (Fig. 4E, Table S3).

This difference could be attributed to “serum-mediated passive dosing” (SMPD Fischer et al. 2018; Lungu-Mitea et al. 2021; Proença et al. 2021). SMPD describes the characteristics of hydrophobic chemicals binding to structural lipids or proteins that are present in certain in vitro test systems, such as serum albumin. Therefore, structural lipids or proteins can act as a sink for hydrophobic compounds and will impact the steady-state equilibrium of freely available concentrations. In our example, we do not employ serum albumin in the BTS preincubation step, but the structural protein within the active or denatured S9 can act as a sink. Within the “W/O S9, W/cof” setups, available exposure concentrations of MET and TPP had been partly lost, and we saw no or reduced effect on NIS inhibition, whereas all S9-containing setups retained some amount of available compound—bound to protein—that was still causing NIS-inhibition effect. Hence, we can also explain the significant differences in effect measures for TPP (log K_ow_ = 4.6; Fig. 4F and Table S1 and S3) via SMPD. Due to its lower hydrophobicity compared to ETX and MET, less available concentration is lost during preincubation to surface binding or evaporation (compare “SM” with “W/O S9, W/cof” for TPP and MET). Still, this effect is overcompensated by the presence of S9 protein (setups “W/S9, W/cof” and “D S9, W/cof”). By binding to the available S9 in the specific BTS setups during the preincubation step, more S9-bound TPP is available post-incubation via SMPD-related partitioning. Consequently, we observed a statistically significant increase in the overall NIS-inhibition effect for the BTS setups that included active and denatured S9 compared to the setup w/cof without BTS (Fig. 4F). Notably, in TPP’s specific case, bioavailability-related effects are rather minuscule compared to MET (Fig. 4E). We conclude that the biotransformation-augmented NIS assay is suitable for chemicals with a log K_ow_ up to approximately 5. However, further testing of compounds within that hydrophobicity margin is necessary to substantiate our results.

### Biotransformation: in silico comparison

To contextualize our experimental results, we employed the in silico BioTransformer 3.0 tool (Wishart et al. 2022); a rule-based, machine-learning algorithm capable of predicting biotransformation metabolites from SMILES inputs and naming the potentially involved enzymatic actors and reactions of detoxification. The computed results (Table S4) corroborate our experimental data. The algorithm predicted the occurrence of phase I and II biotransformation for BPA, DBP, and TCS, involving Cytochrome P450 1A2 (CYP1A2)-mediated hydrolysis reactions, Uridine diphosphate glucuronosyl transferase (UGT)-mediated glucuronidations, Sulfotransferase (SULT)-mediated sulfonations, and Glutathione S-transferase (GST)-mediated conjugations. This prediction corresponds to less NIS inhibition by these compounds after BTS treatment (Fig. 3). Per se, all predicted phase I and II reaction types align with the technical capabilities of the employed BTS, as all necessary cofactors were included in the used BTS (see method section on BTS).

The BioTransfomer algorithm also predicted the metabolization of TPP via CYP2B6-mediated (Cytochrome P450 2B6) dehalogenative photocyclization, which was not mirrored by any decrease of NIS-inhibition activity in our in vitro results (Fig. 4F). This might be caused by comparable NIS-inhibition potential of its metabolites or insufficient TPP metabolization. Notably, human CYP2B6, as BioTransformer outputs relate to human nomenclature, corresponds to CYP2B1 in rats (*Rattus norvegicus*) (Vanoye-Carlo et al. 2015). Rat CYP2B1 expression has been primarily located within the liver, lungs, and brain (Miksys et al. 2000) and has been shown to be inducible via phenobarbital (PB) treatment. However, PB-mediated CYP2B1 induction in rat liver was reportedly 300-fold lower as compared to CYP1A1 induction for identically treated female Wistar rats (Chanyshev et al. 2014). The CYP2B1 activity in the utilized S9 might not be high enough to cause a measurable effect. Indeed, TPP depicted minor but statistically significant differences in effect sizes between the BTS active setup and the non-active “w/o S9 w/cof” control. However, we identified those effects as pertinent rather to matters of bioavailability and not biotransformation.

Also, in line with our in vitro results, the algorithm predicted no biotransformation reactions for the other investigated compounds: ETX, MET, PFOS and RSC. Noteworthy, the prediction was not possible for PCL as the algorithm can only deal with neutral organic compounds and not with ionic substances.

### Biotransformation: comparison to in vivo and in vitro literature results

A direct comparison to the published scientific literature is not possible, as we are the first to develop and employ a biotransformation-augmented NIS-inhibition assay, at least to our knowledge. Nevertheless, literature can be assessed for studies analyzing the same compounds but investigating other endpoints in the context of biotransformation reactions and xenobiotic metabolism. Thereby, one can assess if biotransformation is generally expected for the compounds in question and if our in vitro setup, at least qualitatively, corresponds to in vivo scenarios. We identified several published in vivo and in vitro studies, which addressed the identical compounds.

The three experimentally biotransformed compounds (inactivation–detoxification) BPA, DPB, and TCS, were also reported to be rapidly metabolized and excreted in the wider scientific literature. Effective BPA detoxification and excretion in vivo were reported in humans (Liao and Kannan 2012; Völkel et al. 2002), rats (Kurebayashi 2003), and chimeric mice (Miyaguchi et al. 2015). Moreover, BPA (ant)agonistic effects on the estrogen and androgen receptors were significantly reduced in an in vitro reporter gene assay employing BTS comparable to ours, including Phase I and II detoxification enzymes (Van Vugt-Lussenburg et al. 2018). DBP has been shown to be effectively metabolized and excreted in rats in vivo (Rowland et al. 1977). Similarly, rapid biotransformation was reported for TCS in vivo in several mammalian species (Fang et al. 2010) and fish (Arnot et al. 2018).

We encountered no effect on NIS inhibition by biotransformation for PCL, PFOS, and RSC. Indeed, PCL is known not to be affected by Phase I and II metabolic reactions, but it is directly excreted via urine in humans (Greer et al. 2002; Steinmaus 2016). Similarly, PFOS has been reported to be resistant to xenobiotic metabolism but instead accumulates and persists in mammals, especially humans (Harada et al. 2005). Contrary to our results and also in silico predictions by BioTransformer 3.0, RSC has been reported to be metabolized and excreted as sulfate or glucuronide conjugates in humans (Géniès et al. 2019) and rats (Kim and Matthews’, 1987). Obviously, there is no straight explanation of the discrepancy between our result and in vivo reports, especially in the context of the aforementioned reports not investigating endocrine disruption endpoints but solely focusing on xenobiotic metabolism mechanisms and metabolite identification. It could be hypothesized that, e.g., sulfate conjugates might not significantly alter chemical topology to the extent where steric interactions would modify the NIS symporter inhibition.

Contextually, another study investigated RSC biotransformation in chemico using human EpiSkin-derived S9 fractions and Aroclor-induced, rat-derived S9 in a buffered system (Eilstein et al., 2020). Rat S9 exhibited 66-fold higher UGT activity, enabling RSC glucuronide conjugation, which was absent in EpiSkin S9. Eilstein et al. (2020) employed a 200-fold higher S9 concentration (2 mg/mL) than our study, with similar UDPGA cofactor concentrations (372 µM). Our study required high RSC concentrations (up to 10 mM in the NIS assay and 20 mM during BTS preincubation) to inhibit NIS, contrasting with Eilstein’s use of lower RSC levels (5 µM), resulting in an 8 × 10^5 higher ratio of RSC molarity per S9 mass unit in our setup. Although direct comparison is limited, the absence of RSC biotransformation in EpiSkin S9 at tested concentrations suggests stoichiometric constraints relevant also for our setup, as excess RSC overwhelms the BTS, especially without a cofactor-regeneration system in our BTS formulation.

For future applications, a 24-h BTS preincubation at environmentally relevant concentrations can be used for chemicals metabolized in vivo but showing no biotransformation-related effects in vitro after the 5-h BTS preincubation. Arguably, NIS inhibition is unlikely to be the primary mechanism of action for RSC in disrupting thyroid hormone homeostasis. Instead, other targets, such as thyroid peroxidase (TPO), are more plausible, as interference at much lower micro- and nanomolar concentrations has been demonstrated by us and others (Liu et al. 2024; Van Dingenen et al. 2024).

Finally, for the compounds ETX, MET, and TPP, which are above or slightly below the log K_ow_ ≥ 5 margin, we encountered aberrant NIS-inhibition patterns, which we attributed to bioavailability issues. For ETX, we are unfamiliar with any other literature investigating its biotransformation specifics. MET was reported to be bioactivated by the xenobiotic metabolism machinery, with its metabolite 2,2-bis(p-hydroxyphenyl)-1,1,1-trichloroethane (HPTE) being a potent agonist of the estrogen receptor, both in vivo (Iyer and Makris 2010) and in vitro (Sumida et al. 2001; Van Vugt-Lussenburg et al. 2018). Contrarily, as discussed in previous sections, we only observed effect changes related to SMPD-induced bioavailability rather than bioactivation or biotransformation.

TPP is known to be metabolized and excreted in humans in vivo (Zhao et al. 2019) and in vitro in mouse microsomes (Chen et al. 2022; Chu and Letcher 2019). We discuss above that the concentration of CYP2B1 is likely too low within the employed rat-derived BTS to cause an effect. To what amount this fact can be compared or extrapolated to mouse-derived BTS is unknown, given the generally poor characterization and reporting habits on the enzymatic activity of BTS within most literature (Lungu-Mitea et al., in preparation).

In summary, we conclude that the biotransformation-augmented NIS assay is a proven, reliable technique for evaluating iodide uptake interference in cooperation with a surrogate xenobiotic metabolism system. The findings show that in vitro metabolic capacities could mitigate the inhibitory effects of certain compounds on iodide uptake and that metabolic modification of these compounds and the assessment of downstream endocrine-disrupting effects of metabolites is indeed possible in vitro. Moreover, our experimental results were corroborated by in silico prediction and published in vivo data, at least for the compounds that showed no issues surrounding bioavailability. However, given the limitations with hydrophobic chemicals, we currently suggest implementing the biotransformation-augmented NIS assay only for compounds with a log K_ow_ up to and around five. For more hydrophobic compounds, the assay needs to be re-evaluated in technical terms and scrutinized with a larger set of compounds, including broader physical–chemical parameters.

Noteworthy, we recommend that potential future users of the BTS-augmented NIS assay always include all applied controls (denatured S9, inactive system with cofactors, and the standard NIS method), as these are essential for accurate data interpretation, detecting bioavailability-related effects, and understanding the assay's potential applicability range. Retrospectively, especially for highly hydrophobic compounds, we would recommend the experimenters trying to reproduce our biotransformation-augmented NIS-inhibition assay to add also a 5-h preincubated “standard setup” (“SM”) test group to gain more insights into potential bioavailability issues. The study results emphasize the practicality of supplementing the NIS-inhibition assay with metabolic components to increase its physiological relevance. Enhanced assays are crucial in elucidating the impacts of individual compounds and—potentially—environmental mixtures, ensuring that in vitro evaluations accurately portray real-life hazard scenarios. In addition, the in vitro potential for scalability makes the assay highly advantageous for high-throughput screenings, finally contributing to better environmental and health protection strategies.

### Cross-species relevance of NIS-inhibition assessment

Extrapolating toxicity data from a model species to other species of concern presents significant challenges in chemical hazard assessment, as chemical sensitivities among species can vary dramatically, sometimes by more than a 1000-fold (Doering et al. 2018). Notably, inhibition of the sodium–iodide symporter (NIS) has been associated with adverse health effects across various vertebrates, including mammals, birds, amphibians, and fishes. Based on adverse outcome pathways (AOPs), NIS inhibition is recognized as a molecular initiating event that can lead to various adverse outcomes in different species, such as impaired neurodevelopment in mammals, disrupted swim bladder and eye development in fishes, and altered metamorphosis in amphibians (Noyes et al. 2019).

In this study, we used the SeqAPASS (Sequence Alignment to Predict Across Species Susceptibility) tool developed by the US Environmental Protection Agency (EPA), which allows for the association of chemical susceptibility across species by comparing the amino acid sequences of proteins (US Environmental Protection Agency 2022). Here, we used the human NIS (SLC5A5) sequence as the reference and compared it with the NIS sequences of a vast array of taxa available in SeqAPASS (Figure S8). A high sequence similarity indicates a greater likelihood that the proteins share similar susceptibility to chemical interaction, thus supporting the cross-species relevance of the results from the in vitro models based on human and rat cells. For detailed results of the SeqAPASS predictions, and related discussion and joint interpretation with the relevant studies from the literature based on in silico approaches together with mechanistic and mutation studies, see SI.

In summary, the literature data and SeqAPASS prediction indicate that findings from studies involving human NIS-based models for the identification of THSDCs can be informative for other vertebrates, particularly mammals. This relates to our study using human NIS-transfected cell lines and FRTL-5 rat cells, as well as to other mammalian in vitro models with sensitive NIS-inhibition detection. However, it is important to emphasize that these findings pertain primarily to qualitative cross-species evaluations of chemical susceptibility, namely concerning orthosteric ionic inhibition. Quantitative predictions regarding chemical affinity and inhibition potency, particularly for complex organic compounds that act via allosteric sites, remain uncertain.

## Conclusions

This study demonstrates the applicability of non-radioactive assay for evaluating the inhibitory effects of thyroid hormone-system-disrupting chemicals (THSDCs) on sodium–iodide symporter (NIS) function across different in vitro models. The results from human HEK293T NIS02 (transfected) and rat FRTL-5 (naturally expressing NIS) cell models with SK-reaction-based readouts align well with findings from other in vitro systems and radioactivity-based methodologies. These results underscore the robustness and accessibility of the SK reaction, making it a practical alternative for laboratories lacking the specialized equipment required for handling radioactive materials.

Importantly, the findings highlight the necessity of employing well-characterized in vitro models with adequate NIS-mediated iodide uptake capabilities and high sensitivity to inhibitors, while minimizing confounding effects such as cytotoxicity. This approach ensures a more accurate assessment of NIS inhibition, and we emphasize the importance of also reporting the variability of both NIS inhibition and cytotoxicity parameters across independent experiments for improved reproducibility and transparency. The developed methodology enables the integration of an optional biotransformation step to enhance physiological relevance and predict the in vivo effects of THSDCs with greater accuracy. The study also identifies the impact of metabolic transformation on compounds from diverse use categories, while acknowledging potential limitations of BTS preincubation for highly hydrophobic chemicals.

Overall, this research advances the evaluation of NIS inhibition by diverse compounds, contributing to more accurate chemical risk assessments and the identification of thyroid hormone disruptors. The research is also relevant for medical applications as NIS plays an important role in the diagnostic and therapeutic procedures for diverse types of carcinomas as well as thyroid disorders and NIS inhibition could interfere with the theranostics. The conserved functional domains of the NIS protein across vertebrates reinforce the cross-species relevance of these findings, broadening their applicability in both regulatory and therapeutic frameworks.

## Supplementary Information

Below is the link to the electronic supplementary material.

Supplementary file1 (DOCX 2234 KB)

## Data Availability

Data can be made available upon request.
