# Supplementary material for: Advancing in vitro assessment of iodide uptake inhibition: integrating a novel biotransformation pretreatment step
Source: Arch Toxicol. 2025 May 12;99(7):2783–99. doi: 10.1007/s00204-025-04034-y (PMC12198292; doi:10.1007/s00204-025-04034-y)
Supplement: Supplementary file 1 — Supplementary file1 (DOCX 2234 KB) [file 204_2025_4034_MOESM1_ESM.docx]

**Supplementary Information to:**

Advancing *In Vitro* Assessment of Iodide Uptake Inhibition: Integrating a Novel Biotransformation Pretreatment Step

Puja Kumari, Sebastian Lungu-Mitea, Jiri Novak, Klara Hilscherova^*^

RECETOX, Faculty of Science, Masaryk University, Kotlarska 2, Brno, the Czech Republic

**Contents**

[Material and Methods SI 2](#_Toc192772499)

[Model chemicals selection criteria 2](#_Toc192772500)

[Employed cell lines and culture conditions 2](#_Toc192772501)

[Development of the transfected cell lines 3](#_Toc192772502)

[Characterization of NIS gene and protein expression in the *in vitro* models 4](#_Toc192772503)

[RNA isolation and cDNA Synthesis 4](#_Toc192772504)

[Quantitative Polymerase Chain Reaction (qPCR) 4](#_Toc192772505)

[Protein Expression Analysis 4](#_Toc192772506)

[NIS Inhibition Assay 5](#_Toc192772507)

[Neutral red uptake assay 6](#_Toc192772508)

[CellTiter-Glo assay 6](#_Toc192772509)

[*In silico* biotransformation prediction 6](#_Toc192772510)

[Biotransformation 7](#_Toc192772511)

[Cross-species comparison of SLC5A5 using sequence alignment 8](#_Toc192772512)

[Results and Discussion SI 11](#_Toc192772513)

[NIS biotransformation augmentation 19](#_Toc192772514)

[Cross-species relevance of NIS inhibition assessment 22](#_Toc192772515)

[References 26](#_Toc192772516)

# Material and Methods SI

## Model chemicals selection criteria

Prioritization criteria used for the selection of chemicals in this study included documented *in vivo* effects, relevance for various Thyroid hormone system disruption (THSD) modes of action, inclusion in the EURL ECVAM validation study (Bernasconi et al., 2023), and their significance in human exposure. This study included 21 test compounds (Fig. S1) prioritized within EU H2020 ERGO project (Holbech et al., 2020) to investigate their THSD potential and mechanisms across a spectrum of bioassays and different endpoints. To assess the potential impact of chemical bioavailability on the BTS-augmented *in vitro* system, we have added the compounds etoxazole (ETX) and methoxychlor (MET). ETX (Wang et al., 2018) and MET (US EPA, 2024) were added as reference hydrophobic (log K_ow_ > 5) NIS inhibitors to assess the applicability of the BTS step for hydrophobic chemicals. The chosen compounds encompass a broad spectrum of chemicals relevant for human exposure, including pharmaceuticals, pesticides, industrial substances, environmental pollutants, personal care products, disinfectants, and antiseptics. All chemicals (with at least 98% purity) were obtained from Sigma-Aldrich. Specifications of the chemicals are provided in Table 1 (main article) and Table S2, and their chemical structures are given in Fig. S1.

## Employed cell lines and culture conditions

The spontaneously immortalized rat thyroid follicular cell line FRTL-5 (CVCL_0265) (Bidey et al., 1984) was obtained from Sigma-Aldrich. It was cultured in Coon’s modified Ham’s F12 medium (Sigma Aldrich), which was enriched with 10% fetal bovine serum (FBS, Biosera), L-Glutamine (Sigma Aldrich, 2mM), hydrocortisone (Sigma Aldrich, 10nM), somatostatin (Sigma Aldrich, 10 ng/mL), and Thyroid Stimulating Hormone (TSH; Creative BioMart, 10µg/ml).

The HEK293T (CVCL_0063) (Rothenberg et al., 2010; Mashima et al., 2005) cell line (immortalized human embryonic kidney cells from ATCC) was maintained in DMEM High Glucose medium (Biosera, BioTech) with an addition of 10% FBS (Biosera).

The transfected HEK293T cell lines established within this study (see sections below) were maintained in DMEM High Glucose medium (Biosera) with the addition of 10% FBS (Biosera) and supplemented with 4µg/mL puromycin (Sigma-Aldrich).

The Nthy-ori 3-1 (CVCL_2659) (Rothenberg et al., 2010) (Sigma-Aldrich) is an immortalized human thyroid follicular epithelial cell line that was maintained in RPMI medium (Sigma Aldrich) supplemented with 10% FBS (Biosera).

All the above-listed cell lines were maintained at 37°C with 5% CO_2_. In culture, transfected and non-transfected HEK293T and Nthy-ori 3-1 cells were passaged every three days, whereas FRTL-5 cells were passaged every five days.

## Development of the transfected cell lines

The vector, containing the human NIS gene sequence retrieved from NCBI (NM_000453.3), was produced by Vector Builder, USA (VB200915-1039wfn; Figure S2), purified using NucleoBond Xtra Midi kit (Macherey-Nagel, Germany), and used for the production of lentiviral particles in the Lenti-X™ cell line (TakaraBio Inc., Göteborg, Sweden) using a 2^nd^ generation lentiviral system (Zufferey et al., 1997). For the particle production, an equimolar mixture of the packaging, envelope, and the vector plasmid was used (pMD2.G (Addgene#12259), psPAX2 (Addgene#12260), and VB200915-1039wfn, respectively). These plasmids were combined with polyethyleneimine (PEI, 1mg/ml; Polysciences) in a ratio of 2:1 (plasmids: PEI) in OptiMEM (Sigma-Aldrich). The mixture was applied to the Lenti-X cells and the medium was replaced 3 hours post-transfection. The conditioned lentiviral medium was harvested 48 and 72 hours after transfection, then frozen and stored at -80 ^o^C until further use.

Wild-type HEK293T cells were exposed to the conditioned lentiviral medium supplemented with polybrene (10 µg/ml, Merck). After 24 hours, the medium was replaced with fresh medium containing 2µg/mL puromycin (Sigma) to select for transfected cells. Following puromycin treatment, cells expressing GFP were sorted and isolated using FACS (FACSVerse™ Cell Analyzer, BD Biosciences) to obtain GFP-expressing cell clones (two populations with medium and high GFP signal, and 20 single clone cultures). The transfected HEK293T cell clones expressing GFP were further characterized to confirm successful transfection and expression of the NIS gene.

## Characterization of NIS gene and protein expression in the *in vitro* models

### RNA isolation and cDNA Synthesis

The isolation of RNA samples from the selected *in vitro* models was performed according to the protocols specified in the Macherey-Nagel NucleoSpin TriPrep DNA, RNA, and protein purification reagent kit. RNA concentrations were quantified using a NanoDrop® 1000 spectrophotometer (ThermoFisher Scientific). After converting 1000 ng of RNA to cDNA using the SensiFAST cDNA Synthesis Kit (LabMark) in a PCR-thermocycler (45 cycles) (Biometra, Jena Analytik), a tenfold dilution was performed prior to subsequent procedures.

### Quantitative Polymerase Chain Reaction (qPCR)

Quantitative PCR was conducted using the Kappa SYBR® Fast Universal kit (Merck) on a LightCycler® 480 instrument (Roche) following the manufacturer’s protocol. Specific primer sets for human NIS and rat NIS were designed using the NCBI Primer-BLAST tool and obtained from Elisabeth Pharmacon.

Data analysis was performed using the LightCycler® 480 software with Absolute quantification (2nd derivative, 40 cycles LOQ) and Tm calling (Melt curve genotyping) methods. Each sample was analyzed in technical duplicates, and β-Actin and GAPDH were used as the housekeeping gene for the human NIS gene and β-Actin for the rat NIS gene. Melting curves were generated and compared to positive control samples (Human Total Thyroid RNA, HTT (Invitrogen, Thermo Fisher); Rat Thyroid Total RNA (Zyagen) pooled from four Sprague-Dawley rats (2 males, 2 females 10 weeks old).

### Protein Expression Analysis

Western blotting was performed for the characterization of protein expression in the different in vitro models. Electrophoresis and 12.5% bis-acrylamide gel were prepared using a Mini-PROTEAN® system (BioRad). The electrophoresis was performed at 150 V for 1.5 h.

After electrophoresis, proteins were transferred to Immobilion-E PVDF (Sigma-Aldrich) membranes. The membranes were blocked with 5% w/v skim milk and incubated with primary and secondary antibodies (human NIS IgG antibody, Abcam #ab242007; 1:1000, and Anti-mouse IgG, HRP-linked Antibody, Cellsignal.com #7076S; 1:2500, respectively). Protein detection was performed using a chemiluminescent ECL substrate (Clarity western ECL substrate, Bio-Rad). The resulting images were captured and quantified using a gel documentation system Alliance Q9 (Uvitec).

## NIS Inhibition Assay

- Plate coating was performed using Poly-L-Lysine (PLL): PLL was diluted in ddH_2_O in a 1:3 ratio (1-part PLL and 3 parts ddH_2_O. 50µL of this solution per well was added to 96 well plates and incubated for an hour. After the incubation, plates were washed twice with 100µL ddH_2_O and air-dried for 2 h inside the flowbox to maintain sterility.
- Cell seeding: Cells were seeded at 15,000 cells/well concentration in 200 µL of culture medium. Cells were grown for 48 hours before exposure.
- Exposure of cells to NaI and tested chemical solution: After 48 hours of incubation, the cells were washed twice with 100 µL HBSS buffer. Following washing the cells, the cells were exposed to the test solutions. The test solutions were prepared in glass vials and consisted of uptake buffer (HBSS, 0.5% MeOH (v/v), 5µM NaI) and the test chemical (for maximum tested concentrations, see Table 1), and dilution series was prepared using the uptake buffer with dilution ratio 1:2. From the dilutions, 100 µL of the test solution was added to their respective wells and incubated for two hours in the incubated shaker at 37°C and 120 rpm.
- Plate layout description: The experimental set-up per plate included: N = 12 technical replicates of background cell-free (no iodine uptake) controls, containing the uptake buffer w/o cells; N = 6 technical replicates of negative controls, containing cells in uptake buffer w/o NaI; N = 6 technical replicates of positive controls (used to calculate maximal iodide uptake), containing cells in the uptake buffer. Four rows per plate were allocated for exposure to the test compound dilution series in the uptake buffer. The exposure was conducted in technical triplicates per exposure concentration.
- Post-treatment wash: After 2 h incubation of cells with the test solutions, the cells were washed twice with 100 µL HBSS and blotted on tissue paper to remove traces of iodide present on the plate.
- Iodide calibration: The last row of the plate was used for the iodide calibration consisting of 10 concentrations of NaI (0.009 to 5 µM) as a single technical replicate as the data across many experiments documented excellent reproducibility of the calibration (see Figure S5A).
- Cell lysis and iodide level measurement: The iodide detection and quantification was performed using the Sandell-Kolhoff reaction, based on the catalytic effect of iodide on the reaction between cerium (IV) and arsenic (III). In this reaction, yellow cerium (IV) is reduced to colorless cerium (III) in the presence of arsenic (III) relatively slowly; however, this reduction is accelerated by trace levels of iodide (Waltz et al., 2010). The cells were lysed using 80 µL arsenic solution (25 mM NaAsO_4_, 0.5 M H_2_SO_4_, 0.2 M NaCl; Sigma-Aldrich) and 80 µL ceric solution (25 mM (NH_4_)_4_Ce(SO_4_)_4_, 0.5 M H_2_SO_4_; Sigma-Aldrich). Absorbance was measured after 20 min incubation using a plate reader (Biotek Synergy MX) at 415 nm.

## Neutral red uptake assay

The neutral red uptake assay (NR) was used to assess cell viability according to procedures described in the literature (Borenfreund and Puerner, 1985; Brózman et al., 2020). This assay is based on detecting the uptake and accumulation of the neutral red dye (Sigma-Aldrich) in the lysosomes of viable cells. The exposure was identical to the NIS inhibition detection described above. After the exposure, the cells were washed with PBS and then incubated with neutral red dye for 2 h, allowing live cells to accumulate the dye in their lysosomes.

After incubation, the cells were washed with PBS to remove excess dye. Cells were then lysed using a lysis buffer containing 1% acetic acid in 50% ethanol (v/v). Lysis released the accumulated neutral red from the cells. The amount of released neutral red was measured spectrophotometrically. Absorbance was recorded at 450 nm using a plate reader (Biotek Synergy MX). The recorded absorbance is directly proportional to the number of viable cells. The setup included N = 3 negative controls containing cells with HBSS, N = 3 negative cell-free controls containing HBSS along with 0.5% MeOH (v/v) and 5% NaI (m/v), and N = 3 solvent controls containing cells with HBSS along with 0.5% MeOH (v/v) and 5% NaI (m/v).

## CellTiter-Glo assay

The CellTiter-Glo Luminescent Cell Viability Assay (CTG) (Promega) was employed to orthogonally cross-check the cytotoxicity results obtained from the neutral red assay. This assay is based on detecting ATP levels without the need for repeated washes required in neutral red uptake assay. The assay was performed following the manufacturer’s instructions. Briefly, cells were treated with the chemicals identically as in the NIS inhibition detection described above. Following a 2-hour exposure to the chemicals, the buffer containing iodide, the chemical, and HBSS was carefully removed. Then, 100 µL assay substrate buffer was added, and the buffer was incubated for 15 mins on the shaker for equal distribution. The supernatant was transferred to another plate. Its luminescence was measured using a plate reader (Biotek Synergy mx). Based on the acquired luminescence readings, cell viability was calculated as described in the data analysis section.

## *In silico* biotransformation prediction

The *in silico* BioTransformer 3.0 tool (Wishart et al., 2022), a rule-based machine learning algorithm, was utilized to predict potential metabolites and involved biotransformation reactions based on canonical SMILES inputs. All compounds that were experimentally tested in the biotransformation-augmented NIS inhibition assay were also investigated via BioTransformer. Canonical SMILES were derived from PubChem (https://pubchem.ncbi.nlm.nih.gov/). The following parameters were employed within the interface: Custom Human Multi-Step Transformation; CYP450 Mode – Combined; Step 1 - CYP450, 1 iteration; Step 2 - Phase II, 1 iteration.

## Biotransformation

S9 post-mitochondrial supernatant definition in detail, as provided by the producer (Cat. No. 11-105, Moltox, USA): species: rat; strain: Sprague-Dawley; tissue of origin: liver; age: 5 to 6 weeks; weight: 175-199 g; sex: male; stock total volume: 2.1 mL; buffer system; 0.15 M KCl; storage: -20°C; xenobiotic induction regime: Benzoflavone/Phenobarbital-5,6; stock protein concentration: 36.7 mg/mL; enzymatic activity: EROD induction (Cyp1A1, A2) 80-fold, BROD induction (Cyp2B1, Cyp2B2) 26-fold, PROD induction (Cyp2B1, Cyp2B2) 17-fold, MROD induction (Cyp1A1, A2) 7-fold; further characterization pertinent to the Ames mutagenicity assay (positive for histidine revertant test, positive for benzo(a)pyrene and 2-aminoanthracene bioactivation); BTS-pooling procedures not defined by producer.

10X stock mixtures of BTS components were prepared in 0.5% (v/v) MeOH in HBSS, containing 0.1 mg/mL S9, 50 mM MgCl_2,_ and the following cofactors: 2mM beta-nicotinamide adenine dinucleotide phosphate reduced tetrasodium salt (NADPH, Thermo Scientific™), 5 mM uridine 5′-diphosphoglucuronic acid trisodium salt (UDPGA, Sigma-Aldrich), 20 µM adenosine 3′-phosphate 5′-phosphosulfate triethylammonium salt (PAPS, Sigma-Aldrich), and 2mM L-Glutathione (GSH, Thermo Scientific™). The 10X stock mixtures were prepared in three versions. One containing native S9 fraction (“W/S9, W/cof.”), second with heat-inactivated (denatured) S9 fraction (“D S9, W/cof.”) and third with S9 fraction replaced by HBSS (“W/O S9, W/cof.”). These 10X stock mixtures were used for preparing the BTS pre-incubation reaction mixtures by mixing with the tested chemical (in MeOH) and HBSS in proportion 1:10, and the final MeOH content was 0.5% (v/v).

The tested chemicals in the preincubation reaction mixtures were at the twofold increased (2X) highest non-cytotoxic concentrations causing NIS inhibition (due to a first 1:1 dilution step when applying the reaction mixtures to the NIS inhibition assay plates). After preparing and vortexing all BTS mixture setups, preincubation was conducted in a total volume of 2.2 mL reaction mix per setup in 5 mL amber glass vials for 5 h at 37^o^C.

## Cross-species comparison of SLC5A5 using sequence alignment

SeqAPASS employs a comparative genomics approach to evaluate the functional impact of genetic variations in proteins of interest (including orthologs) across multiple species (LaLone et al., 2016). This tool identifies functionally significant regions within proteins and enables comparisons of chemical-protein interactions across species.

For the analysis, NIS protein sequences were obtained for various vertebrate species, including humans, amphibians, and fishes, from public databases using BLAST+ 2.15.0. These sequences were then aligned using SeqAPASS v7.1 to identify functionally important regions and highlight variations in the amino acid sequences that could influence susceptibility to chemicals. This approach facilitated the prediction of chemical impacts across different species, enhancing the understanding of cross-species relevance of the NIS inhibition detected by human and rat cell-based assays.


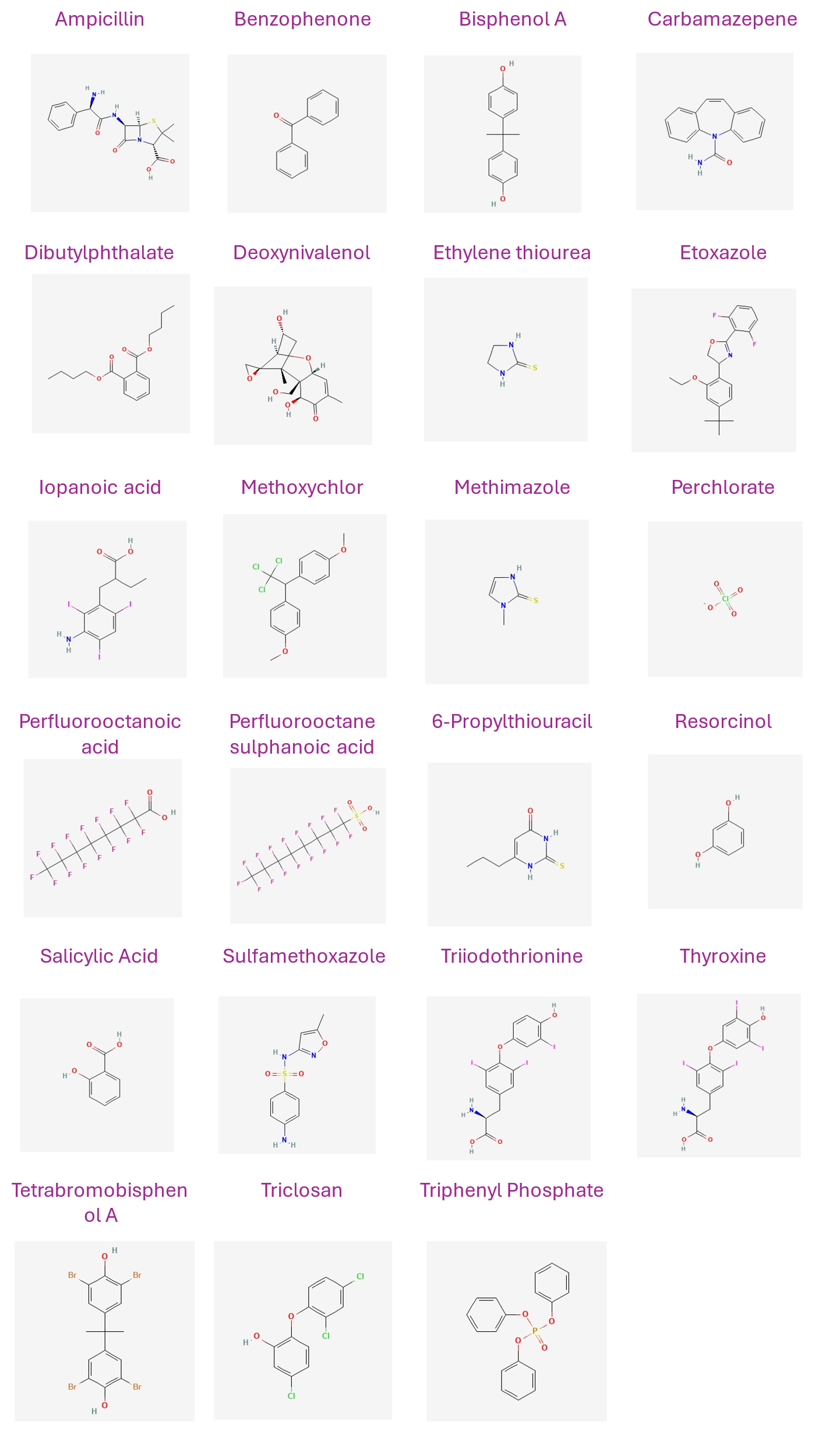


Figure S1. Chemical structures of all chemicals tested in the NIS inhibition assay within this study. Images were obtained from Pubchem https://pubchem.ncbi.nlm.nih.gov/

**
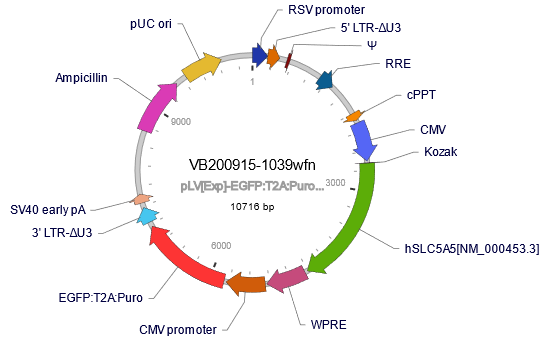
**

Figure S2. Design of the VB200915-1039wfn vector used for the transfection of HEK293T cells. For more details, refer to Vector Builder’s homepage <https://en.vectorbuilder.com/>

#
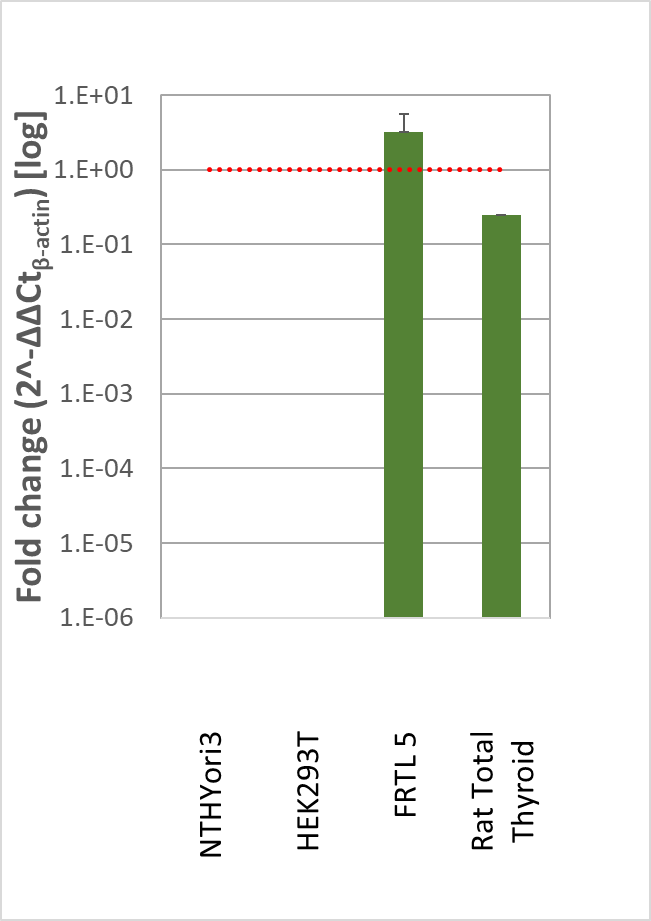
Results and Discussion SI

**B**

**A**


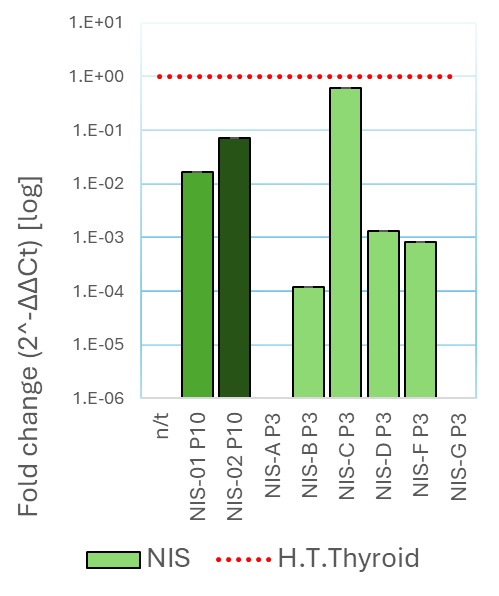


Figure S3. Comparison of SLC5A5 (NIS) gene expression patterns among different transfected cell lines derived from HEK293T compared to untransfected (n/t) cells (A); rat FRTL-5 cell line, non-transfected HEK293T, Nthy-ori 3-1 and rat total thyroid (B). The expression level was compared to the positive control samples (total RNA Human Total Thyroid, HTT (Invitrogen, Thermo Fisher) marked by the red dotted line. NIS expression in rat cell line FRTL-5 is also compared to that in commercial Rat Thyroid Total RNA (Zyagen).


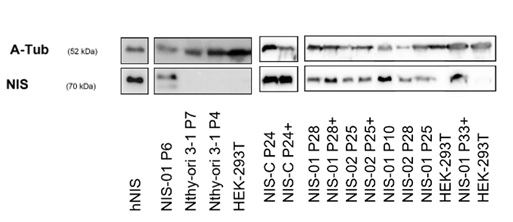


Figure S4. Protein expression of the hNIS along with the housekeeping protein α-Tubulin (A-Tub) with listed molecular weight (kDa) in the transfected HEK293T cells, Nthy-ori 3-1, and non-transfected HEK293T cell models. Bands show the proteins of interest across different cell passages. Passage numbers are indicated (Px). Protein samples from transfected cells cultured without or with (+) 4µg/mL puromycin were compared.

Figure S5. (A): Raw absorbance readouts of iodide calibration curves (used to calculate iodide uptake) across 12 independent experiments, where dots represent the mean and whiskers represent the standard deviation.

*(B): Raw absorbance data in cell-free controls (no-iodide-uptake), HEK293T and Nthy-ori 3-1 cell lines without NIS expression, human NIS transfected HEK293T (NIS 01, NIS 02, NIS C) cell lines and rat FRTL-5 cell line with intrinsic NIS expression. Lower absorbance implies higher iodide uptake. Asterisks mark statistically significant differences in one-way ANOVA followed by Dunnett’s post-hoc test, ****p<0.0001.*

NIS inhibition detection

Figure S6. Comparison between orthogonal neutral red (NR, left column, panels A and C) and Cell Titer Glo (CTG, right column, panels B and D) cytotoxicity assays (red curves) along with NIS inhibition assay (blue curves) in HEK293T NIS 02 cells. Abbreviations, calculations, and illustrations correspond to Figures 1 and 2 in the main article.

Table S1. Physical-chemical properties of the chemicals assessed in the NIS inhibition assay. Data were acquired from PubChem <https://pubchem.ncbi.nlm.nih.gov/>; missing value – no data was available in PubChem.

| **Abbreviation** | **Chemical Name** | **solubility in water (mg/l)** | **log Kow** | **molecular weight (g/mol)** | **Henry‘s law (atm- m^3^/mol)** |
| --- | --- | --- | --- | --- | --- |
| AMP | Ampicillin | 1.01x10^4^ |  | 349.4 |  |
| BPA | Bisphenol A | 120 | 3.32 | 228.29 | 4.0x10^-11^ |
| BP2 | 2,2′-4,4′-Tetrahydroxy Benzophenone | 8821 |  | 246.21 | 3.6x10^-6^ |
| CBZ | Carbamazepine | 18 | 2.45 | 236.27 | 1.1x10^-10^ |
| DBP | Dibutylphthalate | 1.12x10^-2^ | 4.5 | 278.34 | 1.81x10^-6^ |
| DON | Deoxynivalenol | 5.5x10^4^ | -0.71 | 296.31 | 2.0x10^-14^ |
| ETU | Ethylenethiourea |  | -0.66 | 102.16 | 1.4x10^-11^ |
| ETX | Etoxazole | 7.54x10^-2^ | 5.59 | 359.4 | 1.0x10^-7^ |
| IOP | Iopanoic Acid | 348.3 |  | 570.93 |  |
| MET | Methoxychlor | 4x10^-2^ | 5.08 | 345.6 | 2.03x10^-7^ |
| MMI | Methimazole | 2x10^5^ | -0.34 | 114.17 |  |
| PCL | Perchlorate | >20x10^5^ |  | 99.45 |  |
| PFOA | Perfluorooctanoic acid | 3.3x10^3^ |  | 414.07 |  |
| PFOS | Perfluorooctane sulfonate | 56 | -1.08 | 500.13 | 4.34x10^-7^ |
| PTU | Propylthiouracil | 1200 |  | 170.23 | 1.1x10^-9^ |
| RSC | Resorcinol | 7.17x10^5^ | 0.8 | 110.11 | 9.9x10^-11^ |
| SA | Salicylic acid | 2.24x10^3^ | 2.26 | 138.12 |  |
| SMX | Sulfamethoxazole | 610 | 0.89 | 253.28 | 6.4x10^-13^ |
| T3 | Triiodothyronine | 3.958 | 0.93 | 650.97 |  |
| T4 | Thyroxine | 1.62x10^-1^ | 0.16 | 776.87 |  |
| TBBPA | Tetrabromobisphenol A | 1.26 | 4.75 | 543.9 | 2.0x10^-7^ |
| TCS | Triclosan | 10 | 4.76 | 289.5 | 2.1x10^-8^ |
| TPP | Triphenyl phosphate | 1.90 | 4.6 | 326.3 | 3.3x10^-6^ |

Table S2. The summary table comprising the catalog number, CAS number, and IC_50_ values from the NIS inhibition assay (n≥3) and IC_20_ values from the viability assay (NR assay) of all tested chemicals. Metrics are given for all NIS active cell lines tested in this study (HEK293T NIS02, HEK293T NIS C, FRTL-5), along with data acquired from the literature.

*NOTE: * NIS IC_50_ values in the cytotoxic concentration range; † chemical reported to be cytotoxic, for details see the referred study; n.a. no statistically significant effect up to the maximal tested concentration*

| **Cat. No.** | **CAS No.** | **Highest tested**  **conc (µM)** | **Abbrev.** | **Chemicals** | **NIS IC_50_ (µM; mean±SD) / NR IC_20_ (µM; mean±SD)** | | | **Data from literature NIS IC_50_ (µM)** | **Cell lines used in literature** | **Assay Used** | **Reference** |
| --- | --- | --- | --- | --- | --- | --- | --- | --- | --- | --- | --- |
|  |  |  |  |  | **HEK293T NIS02** | **HEK293T NISC** | **FRTL-5** |  |  |  |  |
| 59349-100MG | 69-53-4 | 100 | AMP | Ampicillin | n.e./ n.e. | n.e./ n.e. | n.e./ n.e. |  |  |  |  |
| T16403-25G | 131-55-5 | 100 | BP2 | 2,2′-4,4′-Tetrahydroxy benzophenone | n.e./ n.e. | n.e./ n.e. | n.e. / n.e. |  |  |  |  |
| 239658-50G | 80-05-7 | 500 | BPA | 2,2-Bis(4-hydroxyphenyl)propane (Bisphenol A) | 111 ±75 / n.e. | 309 ±2.5* / *181±21* | 302 ±197* / *364±73* | 112.3 | FRTL-5 | SK | Wu et al., 2016 |
|  |  |  |  |  |  |  |  | 35.5 (IC_20_)^1^ | hNIS-HEK293T-EPA | RAIU | Hallinger et al., 2017 |
|  |  |  |  |  |  |  |  | 24.3±21.6 (IC_20_)^2^ | hNIS-HEK293T-EPA | RAIU | US-EPA, 2024 |
|  |  |  |  |  |  |  |  | n.e. | hNIS-HEK293T-EPA | RAIU | Wang et al., 2018 |
|  |  |  |  |  |  |  |  | 19.5 (IC_20_) ^1^ | hNIS-HEK293T-EPA | RAIU | Wang et al., 2019 |
| 94496-100MG | 298-46-4 | 100 | CBZ | Carbamazepine | n.e. / n.e. | n.e./ n.e. | n.e./ n.e. | n.e. | hNIS-HEK293T-EPA | RAIU | US-EPA, 2024 |
|  |  |  |  |  |  |  |  | n.e. | hNIS-HEK293T-EPA | RAIU | Wang et al., 2019 |
| 524980-100ML | 84-74-2 | 100 | DBP | Dibutylphthalate | 52 ±23 / n.e. | n.e. / *42±13.4* | 92.3 ±11.8 / n.e. | n.e. | hNIS-HEK293T-EPA | SK | Dong et al., 2019 |
|  |  |  |  |  |  |  |  | 63.1 (IC_20_) ^1^ | MCF7hNIS | SK | Dong et al., 2019 |
|  |  |  |  |  |  |  |  | 26.9 | hNIS-HEK293T-EPA | RAIU | US-EPA, 2024 |
|  |  |  |  |  |  |  |  | 191.3 (IC_20_) ^1^ | hNIS-HEK293T-EPA | RAIU | Hallinger et al., 2017 |
|  |  |  |  |  |  |  |  | 26.9 (IC_20_) ^1^ | hNIS-HEK293T-EPA | RAIU | Dong et al., 2019 |
|  |  |  |  |  |  |  |  | n.e. | hNIS-HEK293T-EPA | RAIU | Wang et al., 2018 |
| D0156-5MG | 51481-10-8 | 100 | DON | Deoxynivalenol | n.e. / n.e. | n.e./ n.e. | n.e. / n.e. |  |  |  |  |
| **Cat. No.** | **CAS No.** | **Highest tested**  **conc (µM)** | **Abbrev.** | **Chemicals** | **NIS IC_50_ (µM; mean±SD) / NR IC_20_ (µM; mean±SD)** | | | **Data from literature NIS IC_50_ (µM)** | **Cell lines used in literature** | **Assay Used** | **Reference** |
|  |  |  |  |  | **HEK293T NIS02** | **HEK293T NISC** | **FRTL-5** |  |  |  |  |
| 45531-250MG | 96-45-7 | 100 | ETU | Ethylene thiourea | n.e./ n.e. | n.e./ n.e. | n.e./ n.e. |  |  |  |  |
| 32506-50MG | 153233-91-1 | 100 | ETX | Etoxazole | 7.1 ±4.3 / n.e. | 11.7±8.0^3^/ 28.8±4.6 | 8.05±1.2 /n.e. | 1.2 | hNIS-HEK293T-EPA | RAIU | US-EPA, 2024 |
|  |  |  |  |  |  |  |  | 3.2† | hNIS-HEK293T-EPA | RAIU | Buckalew et al., 2020 |
|  |  |  |  |  |  |  |  | 1.3 | hNIS-HEK293T-EPA | RAIU | Wang et al., 2018 |
|  |  |  |  |  |  |  |  | 1.3† | FRTL-5 | RAIU | Buckalew et al., 2020 |
| 14131-100MG | 96-83-3 | 100 | IOP | Iopanoic acid | n.e. / n.e. | n.e. / n.e. | n.e. / n.e. | n.a | hNIS-HEK293T-EPA | RAIU | US-EPA, 2024 |
| 36161-100MG | 72-43-5 | 500 | MET | Methoxychlor | 11 ±8.7 / n.e. | n.e./ n.e. | 218±4.3 / n.e. | 12.6 (IC_20_)^2^ | hNIS-HEK293T-EPA | RAIU | US-EPA, 2024 |
|  |  |  |  |  |  |  |  | n.e. | hNIS-HEK293T-EPA | RAIU | Wang et al., 2018 |
| M8506-25G | 60-56-0 | 100 | MMI | Methimazole | n.e./ n.e. | n.e./ n.e. | n.e. / n.e. | n.e. | hNIS-HEK293T-EPA | RAIU | US-EPA, 2024 |
|  |  |  |  |  |  |  |  | n.e. | hNIS-HEK293T-EPA | RAIU | Hallinger et al., 2017 |
|  |  |  |  |  |  |  |  | n.e. | hNIS-HEK293T-EPA | RAIU | Wang et al., 2019 |
|  |  |  |  |  |  |  |  | n.e. | hNIS-HEK293T-EPA | RAIU | Buckalew et al., 2020 |
|  |  |  |  |  |  |  |  | n.e. | FRTL-5 | RAIU | Buckalew et al., 2020 |
| **Cat. No.** | **CAS No.** | **Highest tested**  **conc (µM)** | **Abbrev.** | **Chemicals** | **NIS IC_50_ (µM; mean±SD) / NR IC_20_ (µM; mean±SD)** | | | **Data from literature NIS IC_50_ (µM)** | **Cell lines used in literature** | **Assay Used** | **Reference** |
|  |  |  |  |  | **HEK293T NIS02** | **HEK293T NISC** | **FRTL-5** |  |  |  |  |
| 410241-100G | 14797-73-0 | 100 | PCL | Perchlorate | 1.65 ±0.57 / n.e. | 14.9 ±8.3 / n.e. | 5.1 ±4.6 / n.e. | 0.2 (IC_20_) ^1^ | hNIS-HEK293T-EPA | SK | Dong et al., 2019 |
|  |  |  |  |  |  |  |  | 0.2 (IC_20_) ^1^ | MCF7hNIS | SK | Dong et al., 2019 |
|  |  |  |  |  |  |  |  | 0.8 | hNIS-HEK293T-EPA | SK | Dong et al., 2019 |
|  |  |  |  |  |  |  |  | 0.5 | MCF7hNIS | SK | Dong et al., 2019 |
|  |  |  |  |  |  |  |  | 0.1 | FRTL-5 | SK | Waltz et al., 2010 |
|  |  |  |  |  |  |  |  | 0.3 | FRTL-5 | SK | Wu et al., 2016 |
|  |  |  |  |  |  |  |  | 1.56 | HeLa transfected with hNIS | RAIU | Concilio et al., 2020. |
|  |  |  |  |  |  |  |  | 0.2 (IC_20_) ^1^ | hNIS-HEK293T-EPA | RAIU | Hallinger et al., 2017 |
|  |  |  |  |  |  |  |  | 0.1 (IC_20_) ^1^ | hNIS-HEK293T-EPA | RAIU | Dong et al., 2019 |
|  |  |  |  |  |  |  |  | 0.53 | hNIS-HEK293T-EPA | RAIU | Dong et al., 2019 |
|  |  |  |  |  |  |  |  | 0.2 | FRTL-5 | RAIU | Buckalew et al., 2020 |
|  |  |  |  |  |  |  |  | 0.14 | FRTL-5 | RAIU | Waltz et al., 2010 |
|  |  |  |  |  |  |  |  | 4.59 | HeLa transfected with whale NIS | RAIU | Concilio et al., 2020 |
|  |  |  |  |  |  |  |  | 0.08 | HeLa transfected with zebrafish NIS | RAIU | Concilio et al., 2020 |
| 33824-100MG | 335-67-1 | 100 | PFOA | Perfluorooctanoic  acid | n.e. / n.e. | n.e./ n.e. | n.e. / n.e. | n.e. | hNIS-HEK293T-EPA | RAIU | US-EPA, 2024 |
|  |  |  |  |  |  |  |  | 75.9 | hNIS-HEK293T-EPA | RAIU | Buckalew et al., 2020 |
|  |  |  |  |  |  |  |  | n.e. | hNIS-HEK293T-EPA | RAIU | Wang et al., 2018 |
|  |  |  |  |  |  |  |  | 25.1 | FRTL-5 | RAIU | Buckalew et al., 2020 |
| 77282-10G | 2795-39-3 | 100 | PFOS | Perfluorooctane sulfonate | 30.9 ±21.7 / n.e. | n.e. / n.e. | 9.31 ±4.34 / n.e. | 20.5 | hNIS-HEK293T-EPA | RAIU | US-EPA, 2024 |
|  |  |  |  |  |  |  |  | 1.3† | hNIS-HEK293T-EPA | RAIU | Buckalew et al., 2020 |
|  |  |  |  |  |  |  |  | 16.6 | hNIS-HEK293T-EPA | RAIU | Wang et al., 2018 |
|  |  |  |  |  |  |  |  | 17 | hNIS-HEK293T-EPA | RAIU | Wang et al., 2019 |
|  |  |  |  |  |  |  |  | 0.4† | FRTL-5 | RAIU | Buckalew et al., 2020 |
| **Cat. No.** | **CAS No.** | **Highest tested**  **conc (µM)** | **Abbrev.** | **Chemicals** | **NIS IC_50_ (µM; mean±SD) / NR IC_20_ (µM; mean±SD)** | | | **Data from literature NIS IC_50_ (µM)** | **Cell lines used in literature** | **Assay Used** | **Reference** |
|  |  |  |  |  | **HEK293T NIS02** | **HEK293T NISC** | **FRTL-5** |  |  |  |  |
| P3755-25G | 51-52-5 | 100 | PTU | 6-propylthiouracil | n.e. / n.e. | n.e. / n.e. | n.e. / n.e. | n.e. | hNIS-HEK293T-EPA | RAIU | Hallinger et al., 2017 |
|  |  |  |  |  |  |  |  | n.e. | hNIS-HEK293T-EPA | RAIU | Buckalew et al., 2020 |
| 307521-100G | 108-46-3 | 10000 | RSC | Resorcinol | 4057±2048 / n.e. | n.e./ n.e. | 4709.5 ±562 / n.e. | n.e. | hNIS-HEK293T-EPA | RAIU | Wang et al., 2019 |
| S5922-100G | 69-72-7 | 100 | SA | Salicylic acid | n.e. / n.e. | n.e. / n.e. | n.e. / n.e. |  |  |  |  |
| S7507-10G | 723-46-6 | 100 | SMX | Sulfamethoxazol | n.e. / n.e. | n.e. / n.e. | n.e. / n.e. |  |  |  |  |
| T6397-250MG | 6893-02-3 | 100 | T3 | 3,3′,5-Triiodo-L-  thyronine | n.e. / n.e. | n.e. / n.e. | n.e. / n.e. | n.e. | hNIS-HEK293T-EPA | RAIU | US-EPA, 2024 |
| T2376-500MG | 51-48-9 | 100 | T4 | 3,3′,5,5″-Tetraiodo-L-thyronine | n.e. / n.e. | n.e. / n.e. | n.e. / n.e. |  |  |  |  |
| 330396-100G | 79-94-7 | 100 | TBBPA | Tetrabromo  bisphenol A | 30.1 ±11.8* / *66.8±32.2* | 54 ±27.1* / *57.6±40.8* | 91.8 ±10.8 * / *33.4±17.1* | 91.1 † | hNIS-HEK293T-EPA | RAIU | US-EPA, 2024 |
| PHR1338-1G | 3380-34-5 | 100 | TCS | Triclosan | 18.5 ±9.9^3^ / *45.9±4* | 34.8 ±23.5* / *36.3±12.8* | 39.3 ±18.9* / 38.9±0.3 | 21.3 | FRTL-5 | SK | Wu et al., 2016 |
|  |  |  |  |  |  |  |  | 16.3±7.4 † | hNIS-HEK293T-EPA | RAIU | US-EPA, 2024 |
|  |  |  |  |  |  |  |  | 2.6 (IC_20_) † ^[[1]](#footnote-2)^ | hNIS-HEK293T-EPA | RAIU | Hallinger et al., 2017 |
|  |  |  |  |  |  |  |  | 7.8† | hNIS-HEK293T-EPA | RAIU | Wang et al., 2018 |
| 241288-50G | 115-86-6 | 100 | TPP | Triphenyl phosphate | 42.8 ±18.9 / n.e. | n.e. / n.e. | 92.6 ±24.1 / n.e. | 50† | hNIS-HEK293T-EPA | RAIU | US-EPA, 2024 |

## NIS biotransformation augmentation

Figure S7. Effects on HEK293T NIS02 cellular viability (measured by NR - neutral red assay) within the NIS inhibition assay augmented with Biotransformation system (BTS). Abbreviations of the chemicals are explained in Table 1 (main article) and S1. The BTS setups were as follows: W/O S9 and W/cof (green curve), representing chemicals treated without the S9 fraction but with all cofactors added; D S9, W/cof (orange curve), where chemicals were treated with denatured S9 and all cofactors added; W/S9, W/cof (purple curve), indicating treatment with both S9 fraction and cofactors. Graphical data representation elements correspond to the information given in Figures 3 and 4 from three independent experiments.

*Table S3. NIS inhibition IC_20_ values and their ratios for all setups used in biotransformation (BTS) experiments, derived from three independent experiments for chemicals affected by BTS treatment (with statistically significant differences determined by 2-way mixed model ANOVA). In cases where IC_20_ values fall beyond the tested concentration range, they are reported as "<x", or ">x", where x represents the highest concentration tested. "n.a." indicates that data were not available (confidence intervals could not be computed). The BTS setups were as follows: “W/O S9, W/cof”, without the S9 fraction but with all cofactors added; “D S9, W/cof”, with denatured S9 fraction and cofactors; “W/S9, W/cof”, S9 fraction and cofactors were added (fully active system); and SM, the standard method for the NIS inhibition assay.*

| Abb. | Chemicals | IC_20_ W/O S9, W/cof [µM] | IC_20_ D S9, W/cof [µM] | IC_20_ W/S9, W/cof [µM] | IC_20_ SM  [µM] | IC_20_ (W/S9, W/cof)/ IC_20_ (D S9, W/cof) [µM] |
| --- | --- | --- | --- | --- | --- | --- |
| BPA | Bisphenol A | 4.9±4.7 | 2.38±5 | 14.9 ±2.2 | 4.7±6 | 6.26 |
| DBP | Dibutylphthalate | 6.24±3.85 | 11.1±3.5 | >100 | 5.57±4.6 | >9 |
| ETX | Etoxazole | 5.17±2.3 | 3.45±1.74 | 2.45±1.45 | 0.61±n.a. | 0.71 |
| MET | Methoxychlor | >500 | 20.7±19.5 | <4 | <4 | ~0.2 |
| TCS | Triclosan | 1.02±0.82 | 1.07±0.65 | 5.32±4.83 | 1.78±1.12 | 4.99 |
| TPP | Triphenylphosphate | 17.5±7.2 | 9.97±3.36 | 7.11±2.63 | 7.93±4.47 | 0.71 |

Table S4. Summary of BioTransformer 3.0 in silico biotransformation predictions pertinent to the primarily involved enzymatic actors. For perchlorate (PCL), the prediction was not realized due to the physical-chemical restrictions of the in silico model.

| **Compound** | **Abbreviation** | **Smiles** | **In silico biotransformation** | **Primarily involved mechanisms** |
| --- | --- | --- | --- | --- |
| Bisphenol A | BPA | Oc1ccc(cc1)C(c2ccc(O)cc2)(C)C | YES | CYP1A2, UDPG, COM |
| Dibutyl phthalate | DBP | CCCCOC(=O)c1ccccc1C(=O)OCCCC | YES | CYP1A2, UDPG, SULT |
| Etoxazole | ETX | CCOc1cc(ccc1C2COC(=N2)c3c(cccc3F)F)C(C)(C)C | NO |  |
| Methoxychlor | MET | ClC(Cl)(Cl)C(c1ccc(OC)cc1)c2ccc(OC)cc2 | NO |  |
| Perchlorate | PCL | [O-][Cl+3]([O-])([O-])[O-] | - |  |
| Perfluorooctane sulfonate | PFOS | FC(F)(C(F)(F)S(=O)(=O)O)C(F)(F)C(F)(F)C(F)(F)C(F)(F)C(F)(F)C(F)(F)F | NO |  |
| Resorcinol | RSC | c1cc(cc(c1)O)O | NO |  |
| Triclosan | TCS | Clc2cc(Cl)ccc2Oc1ccc(Cl)cc1O | YES | CYP1A2, UDPG, GSH |
| Triphenyl phosphate | TPP | O=P(Oc1ccccc1)(Oc2ccccc2)Oc3ccccc3 | YES | CYP2B6 |

## Cross-species relevance of NIS inhibition assessment

The SeqAPASS analysis was performed at levels 1 and 2, with level 1 representing cross-taxa amino acid sequence alignment for the entire NIS protein and level 2 relating to the NIS solute-binding domain. Especially within mammals and common vertebrate test species sequence similarity scored high (Figure S8, Table S5) and, thus, chemical susceptibility across taxa is likely (LaLone et al., 2016). The SeqAPASS analysis of key proteins involved in THSD-related MIEs was also shown in Haigis et al. (2023). In this work, NIS inhibition was identified as an MIE with significant cross-species relevance not only due to the relatively high level of NIS protein similarity but also based on empirical evidence of similar effects of inorganic NIS inhibitors on TH levels in diverse vertebrate model species *in vivo.* For mammals, the level of confidence for NIS inhibition in relation to THSD was considered high, and moderate in the case of fish and amphibians, respectively. For birds and reptiles, the confidence was lower due to the absence of empirical evidence supporting results from the SeqAPASS analysis.

Similarly, a study conducted by Concilio et al. (2020) utilised EMBOSS Needle Pairwise Sequence Alignment for prominent vertebrate species. It revealed 84 - 98% similarity of amino acid sequence of whole protein across seven mammalian species compared to human NIS. Lower amino acid sequence similarity was recorded in fish species (70 - 72%; *D. rerio*, *C. harengus*), birds (75%; *H. leucocephalus*), amphibia (76%; *X. laevis*), and reptiles (53%, *P. sinensis*). Out of the sequenced species, the authors transduced NIS genes from Humans, Zebrafish (*D. rerio*), and Minke whale (*B. acutorostrata*), and assessed them for inhibition by a set of inorganic anions. While NIS was susceptible to inhibition by the inorganic anions in all three species, inhibition potency varied across species. For example, PCL depicted an IC_50_ of 4.6 µM in Minke whales, 1.6 µM in Humans, and 0.08 µM in Zebrafish *in vitro* systems. In general terms, for the tested set of inorganic ions, inhibition potencies coincided with a greater magnitude between Humans and Mink whales (sequence similarity 91%), as compared to Zebrafish. Moreover, Ravera et al. (2022) unveiled that canonical residues within the NIS solute-binding domain, which are paramount to the NIS-to-substrate interaction, are fully conserved and functional across prominent vertebrate test species. Therefore, we conclude that our SeqAPASS analysis is corroborated by the literature.

Nevertheless, it is noteworthy that amino acid sequence similarity and, thus, associated chemical susceptibility do not directly translate to substrate affinity and the potency of the inhibitory effect. Several structural mutagenesis studies have revealed that the substitution of single canonical residues within the NIS solute-binding domain can at least impair or completely halt NIS activity (Ferrandino et al., 2016; Ravera et al., 2022; Wu et al., 2008). Additionally, even for evolutionary, more closely related NIS orthologs, differences in cellular NIS localisation have been demonstrated to impact overall symporter activity (Dayem et al., 2008; Josefsson et al., 2002; Petersen et al., 2022). Finally, all the above-mentioned studies predominantly address NIS impairment through potential inhibitors at the orthosteric site. Contrarily, even the ionic inhibitor PCL has also been reported to exert its effects through a secondary allosteric site (Llorente-Esteban et al., 2020). Furthermore, preliminary evidence suggests that non-ionic, complex organic chemicals, like those examined in our study, primarily inhibit NIS activity via allosteric sites (Lecat-Guillet et al., 2008; Lindenthal et al., 2009). The exact locations of these allosteric sites within the NIS polypeptide chain and their subsequent implications for NIS function remain to be elucidated.


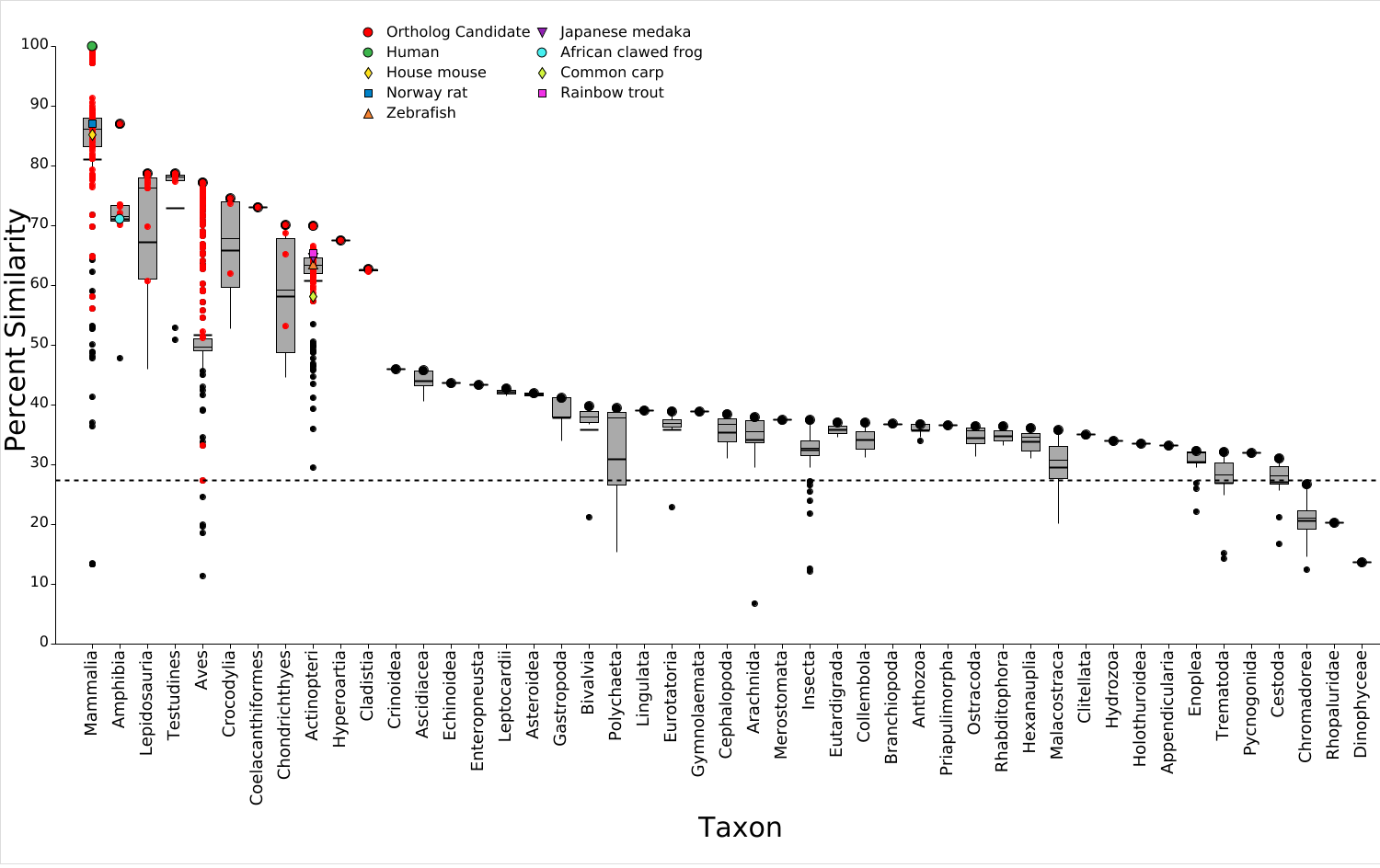


Figure S8. Percent similarity of NIS protein binding domain (solute binding domain) based on the alignment of the primary amino acid sequence by SeqAPASS (level 2) across the available protein sequence from different organisms. The susceptibility cut-off (27.3%) discriminates orthologs from other similar proteins in the analysis.

Table S5 Summary of SeqAPASS analysis of NIS protein shows significant similarity across common animal model species when comparing both whole protein (Level 1) and the solute-binding domain- SLC5sbd_NIS (Level 2)

| **Species** | **Protein** | **Level 1 Susceptible (Y/N)** | **Level 2 Susceptible  (Y/N)** |
| --- | --- | --- | --- |
| Human | sodium/iodide cotransporter | Y | Y |
| Norway rat | RecName: Full=Sodium/iodide cotransporter; Short=Na(+)/I(-) cotransporter; AltName: Full=Sodium-iodide symporter; Short=Na(+)/I(-) symporter; AltName: Full=Solute carrier family 5 member 5 | Y | Y |
| House mouse | RecName: Full=Sodium/iodide cotransporter; Short=Na(+)/I(-) cotransporter; AltName: Full=Sodium-iodide symporter; Short=Na(+)/I(-) symporter; AltName: Full=Solute carrier family 5 member 5 | Y | Y |
| African clawed frog | solute carrier family 5 member 5 L homeolog | Y | Y |
| Rainbow trout | sodium/iodide cotransporter isoform X1 | Y | Y |
| Japanese medaka | sodium/iodide cotransporter isoform X1 | Y | Y |
| Zebrafish | sodium/iodide cotransporter | Y | Y |
| Common carp | hypothetical protein cypCar_00021973 | Y | Y |

# References

Bernasconi, C., Bartnicka, J., Asturiol, D., Bowe, G., Langezaal, I., Coecke, S., Kienzler, A., Liska, R., Milcamps, A., Munoz Pineiro, A., Pistollato, F., Whelan, M., European Commission. Joint Research Centre., 2023. Validation of a battery of mechanistic methods relevant for the detection of chemicals that can disrupt the thyroid hormone system. https://doi.org/10.2760/862948

Bidey, S.P., Chiovato, L., Day, A., Turmaine, M., Gould, R.P., Ekins, R.P., Marshall, N.J., 1984. Evaluation of the rat thyroid cell strain FRTL-5 as an in-vitro bioassay system for thyrotrophin. J. Endocrinol. 101, 269-NP. https://doi.org/10.1677/joe.0.1010269

Borenfreund, E., Puerner, J.A., 1985. Toxicity determined in vitro by morphological alterations and neutral red absorption. Toxicol. Lett. 24, 119–124. https://doi.org/10.1016/0378-4274(85)90046-3

Brózman, O., Novák, J., Bauer, A.K., Babica, P., 2020. Airborne PAHs inhibit gap junctional intercellular communication and activate MAPKs in human bronchial epithelial cell line. Environ. Toxicol. Pharmacol. 79, 103422. https://doi.org/10.1016/j.etap.2020.103422

Buckalew, A.R., Wang, J., Murr, A.S., Deisenroth, C., Stewart, W.M., Stoker, T.E., Laws, S.C., 2020. Evaluation of potential sodium-iodide symporter (NIS) inhibitors using a secondary Fischer rat thyroid follicular cell (FRTL-5) radioactive iodide uptake (RAIU) assay. Arch. Toxicol. 94, 873–885. https://doi.org/10.1007/s00204-020-02664-y

Concilio, S.C., Zhekova, H.R., Noskov, S.Y., Russell, S.J., 2020. Inter-species variation in monovalent anion substrate selectivity and inhibitor sensitivity in the sodium iodide symporter (NIS). PLOS ONE 15, e0229085. https://doi.org/10.1371/journal.pone.0229085

Dayem, M., Basquin, C., Navarro, V., Carrier, P., Marsault, R., Chang, P., Huc, S., Darrouzet, E., Lindenthal, S., Pourcher, T., 2008. Comparison of expressed human and mouse sodium/iodide symporters reveals differences in transport properties and subcellular localization. J. Endocrinol. 197, 95–109. https://doi.org/10.1677/JOE-07-0455

Dong, H., Atlas, E., Wade, M.G., 2019. Development of a non-radioactive screening assay to detect chemicals disrupting the human sodium iodide symporter activity. Toxicol. In Vitro 57, 39–47. https://doi.org/10.1016/j.tiv.2019.01.021

Ferrandino, G., Nicola, J.P., Sánchez, Y.E., Echeverria, I., Liu, Y., Amzel, L.M., Carrasco, N., 2016. Na+ coordination at the Na2 site of the Na+/I− symporter. Proc. Natl. Acad. Sci. 113, E5379–E5388. https://doi.org/10.1073/pnas.1607231113

Haigis, A.-C., Vergauwen, L., LaLone, C.A., Villeneuve, D.L., O’Brien, J.M., Knapen, D., 2023. Cross-species applicability of an adverse outcome pathway network for thyroid hormone system disruption. Toxicol. Sci. 195, 1–27. https://doi.org/10.1093/toxsci/kfad063

Hallinger, Daniel R., Murr, A.S., Buckalew, A.R., Simmons, S.O., Stoker, T.E., Laws, S.C., 2017. Development of a screening approach to detect thyroid disrupting chemicals that inhibit the human sodium iodide symporter (NIS). Toxicol. In Vitro 40, 66–78. https://doi.org/10.1016/j.tiv.2016.12.006

Josefsson, M., Grunditz, T., Ohlsson, T., Ekblad, E., 2002. Sodium/iodide-symporter: distribution in different mammals and role in entero-thyroid circulation of iodide. Acta Physiol. Scand. 175, 129–137. https://doi.org/10.1046/j.1365-201X.2002.00968.x

LaLone, C.A., Villeneuve, D.L., Lyons, D., Helgen, H.W., Robinson, S.L., Swintek, J.A., Saari, T.W., Ankley, G.T., 2016. Sequence alignment to predict across species susceptibility (seqapass): A web-based tool for addressing the challenges of cross-species extrapolation of chemical toxicity. Toxicol. Sci. 153, 228–245. https://doi.org/10.1093/toxsci/kfw119

Lecat-Guillet, N., Merer, G., Lopez, R., Pourcher, T., Rousseau, B., Ambroise, Y., 2008. Small-Molecule Inhibitors of Sodium Iodide Symporter Function. ChemBioChem 9, 889–895. https://doi.org/10.1002/cbic.200700682

Lindenthal, S., Lecat-Guillet, N., Ondo-Mendez, A., Ambroise, Y., Rousseau, B., Pourcher, T., 2009. Characterization of small-molecule inhibitors of the sodium iodide symporter. J. Endocrinol. 200, 357–365. https://doi.org/10.1677/JOE-08-0246

Llorente-Esteban, A., Manville, R.W., Reyna-Neyra, A., Abbott, G.W., Amzel, L.M., Carrasco, N., 2020. Allosteric regulation of mammalian Na+/I− symporter activity by perchlorate. Nat. Struct. Mol. Biol. 27, 533–539. https://doi.org/10.1038/s41594-020-0417-5

Mashima, T., Oh-hara, T., Sato, S., Mochizuki, M., Sugimoto, Y., Yamazaki, K., Hamada, J., Tada, M., Moriuchi, T., Ishikawa, Y., Kato, Y., Tomoda, H., Yamori, T., Tsuruo, T., 2005. p53-Defective Tumors With a Functional Apoptosome-Mediated Pathway: A New Therapeutic Target. JNCI J. Natl. Cancer Inst. 97, 765–777. https://doi.org/10.1093/jnci/dji133

Petersen, A.M., Small, C.M., Yan, Y.-L., Wilson, C., Batzel, P., Bremiller, R.A., Buck, C.L., von Hippel, F.A., Cresko, W.A., Postlethwait, J.H., 2022. Evolution and developmental expression of the sodium–iodide symporter (, slc5a5) gene family: Implications for perchlorate toxicology. Evol. Appl. 15, 1079–1098. https://doi.org/10.1111/eva.13424

Ravera, S., Nicola, J.P., Salazar-De Simone, G., Sigworth, F.J., Karakas, E., Amzel, L.M., Bianchet, M.A., Carrasco, N., 2022. Structural insights into the mechanism of the sodium/iodide symporter. Nature 612, 795–801. https://doi.org/10.1038/s41586-022-05530-2

Rothenberg, S.M., Mohapatra, G., Rivera, M.N., Winokur, D., Greninger, P., Nitta, M., Sadow, P.M., Sooriyakumar, G., Brannigan, B.W., Ulman, M.J., Perera, R.M., Wang, R., Tam, A., Ma, X.-J., Erlander, M., Sgroi, D.C., Rocco, J.W., Lingen, M.W., Cohen, E.E.W., Louis, D.N., Settleman, J., Haber, D.A., 2010. A Genome-Wide Screen for Microdeletions Reveals Disruption of Polarity Complex Genes in Diverse Human Cancers. Cancer Res. 70, 2158–2164. https://doi.org/10.1158/0008-5472.CAN-09-3458

US EPA, 2024. ToxCast CompTox Chemicals Dashboard Version 2.4.1 [WWW Document]. URL https://comptox.epa.gov/dashboard/ (accessed 10.14.24).

Waltz, F., Pillette, L., Ambroise, Y., 2010. A nonradioactive iodide uptake assay for sodium iodide symporter function. Anal. Biochem. 396, 91–95. https://doi.org/10.1016/j.ab.2009.08.038

Wang, J., Hallinger, D.R., Murr, A.S., Buckalew, A.R., Lougee, R.R., Richard, A.M., Laws, S.C., Stoker, T.E., 2019. High-throughput screening and chemotype-enrichment analysis of ToxCast phase II chemicals evaluated for human sodium-iodide symporter (NIS) inhibition. Environ. Int. 126, 377–386. https://doi.org/10.1016/j.envint.2019.02.024

Wang, J., Hallinger, D.R., Murr, A.S., Buckalew, A.R., Simmons, S.O., Laws, S.C., Stoker, T.E., 2018. High-Throughput Screening and Quantitative Chemical Ranking for Sodium-Iodide Symporter Inhibitors in ToxCast Phase i Chemical Library. Environ. Sci. Technol. 52, 5417–5426. https://doi.org/10.1021/acs.est.7b06145

Wishart, D.S., Tian, S., Allen, D., Oler, E., Peters, H., Lui, V.W., Gautam, V., Djoumbou-Feunang, Y., Greiner, R., Metz, T.O., 2022. BioTransformer 3.0—a web server for accurately predicting metabolic transformation products. Nucleic Acids Res. 50, W115–W123. https://doi.org/10.1093/nar/gkac313

Wu, S.-L., Ho, T.-Y., Liang, J.-A., Hsiang, C.-Y., 2008. Histidine residue at position 226 is critical for iodide uptake activity of human sodium/iodide symporter. J. Endocrinol. 199, 213–219. https://doi.org/10.1677/JOE-08-0249

Wu, Y., Beland, F.A., Fang, J.L., 2016. Effect of triclosan, triclocarban, 2,2’,4,4’-tetrabromodiphenyl ether, and bisphenol A on the iodide uptake, thyroid peroxidase activity, and expression of genes involved in thyroid hormone synthesis. Toxicol. In Vitro 32, 310–319. https://doi.org/10.1016/j.tiv.2016.01.014

Zufferey, R., Nagy, D., Mandel, R.J., Naldini, L., Trono, D., 1997. Multiply attenuated lentiviral vector achieves efficient gene delivery in vivo. Nat. Biotechnol. 15, 871–875. https://doi.org/10.1038/nbt0997-871

1. 1 IC_20_ values reported here correspond to EC_80_ values in publications from ToxCast data (Dong et al., 2019; Hallinger et al., 2017; Wang et al., 2019, 2018), as they define 100% as no effect. Hence, 80% response represents 20% reduction in the activity, aligning with IC_20_.

   ^2^ Although BPA and MET are described as inactive in the ToxCast because they did not reach effect cut-off (56%) used there, their dose-response data clearly show both chemicals are active although the inhibition did not reach 50% effect, so we provide IC_20_ calculated from the available data

   ^3^ NIS IC_50_ was not in the cytotoxic concentration range, but cytotoxicity occurred at greater exposure levels (see relevant charts in Fig. 1, 2) [↑](#footnote-ref-2)
